# Identification of Chemosensory Genes Based on the Antennal Transcriptomic Analysis of *Plagiodera versicolora*

**DOI:** 10.3390/insects13010036

**Published:** 2021-12-29

**Authors:** Xiaolong Liu, Na Tong, Zheran Wu, Yang Li, Meiqi Ma, Pei Liu, Min Lu

**Affiliations:** State Key Laboratory of Biocatalysis and Enzyme Engineering, School of Life Sciences, Hubei University, Wuhan 430062, China; 2017202032@njau.edu.cn (X.L.); hubeitn@163.com (N.T.); wuzheran99@163.com (Z.W.); 15727131591@163.com (Y.L.); mmq736170@163.com (M.M.); liupei795939@163.com (P.L.)

**Keywords:** *Plagiodera versicolora*, antennal transcriptome, odorant binding proteins, odorant receptors

## Abstract

**Simple Summary:**

In this study, we conducted a transcriptome analysis of adult antennae in *Plagiodera versicolora* (Coleoptera: Chrysomelidae) and identified a total of 98 candidate chemosensory genes, encoding 40 odorant receptors (ORs), 7 ionotropic receptors (IRs), 13 gustatory receptors (GRs), 10 chemosensory proteins (CSPs), 24 odorant binding proteins (OBPs), and 4 sensory neuron membrane proteins (SNMPs). The tissue expression profiles showed that almost all PverOBPs and PverORs were highly expressed in the antennae. In addition, the results revealed that PverOBP10, PverOBP12, PverOBP18, PverOR24, and PverOR35 showed female-biased expression profiles, indicating that these receptors may be involved in some female-specific behaviors such as oviposition site seeking. This work greatly promotes the understanding of the olfactory system and will help provide insight for functional studies of the chemoreception mechanism in *P. versicolora*.

**Abstract:**

Insects can sense surrounding chemical signals by their accurate chemosensory systems. This system plays a vital role in the life history of insects. Several gene families participate in chemosensory processes, including odorant receptors (ORs), ionotropic receptors (IRs), gustatory receptors (GRs), chemosensory proteins (CSPs), odorant binding proteins (OBPs), and sensory neuron membrane proteins (SNMPs). *Plagiodera versicolora* (Coleoptera: Chrysomelidae), is a leaf-eating forest pest found in salicaceous trees worldwide. In this study, a transcriptome analysis of male and female adult antennae in *P. versicolora* individuals was conducted, which identified a total of 98 candidate chemosensory genes including 40 ORs, 7 IRs, 13 GRs, 10 CSPs, 24 OBPs, and 4 SNMPs. Subsequently, the tissue expression profiles of 15 *P. versicolora* OBPs (PverOBPs) and 39 ORs (PverORs) were conducted by quantitative real-time PCR. The data showed that almost all PverOBPs and PverORs were highly expressed in the male and female antennae. In addition, several OBPs and ORs (PverOBP10, PverOBP12, PverOBP18, PverOR24, and PverOR35) had higher expression levels in female antennae than those in the male antennae, indicating that these genes may be taking part in some female-specific behaviors, such as find mates, oviposition site, etc. This study deeply promotes further understanding of the chemosensory system and functional studies of the chemoreception genes in *P. versicolora*.

## 1. Introduction

Insects depend on a complex chemosensory system to find mates, oviposition sites, foods, and to evade predators or toxic compounds [1]. Generally, the antennae, proboscis, legs, and labial palps are the main chemosensory organs in insects [2]. The chemosensory system involves several different types of genes, including odorant receptors (ORs), odorant binding proteins (OBPs), chemosensory proteins (CSPs), gustatory receptors (GRs), ionotropic receptors (IRs), and sensory neuron membrane proteins (SNMPs) [3,4,5,6]. The peripheral level of the insect chemosensory system general includes several major steps; after entering into the sensilla lymph through pores on the sensilla wall, the hydrophobic odorant molecules are bound by soluble olfactory proteins (e.g., OBPs and CSPs) in the sensilla lymph. This complex is subsequently transported to corresponding chemosensory receptors (ORs, GRs, and IRs proteins), which finally induces an action potential and guides insects’ behavior [4,7,8,9,10]. For example, ApisOBP3 and ApisOBP7 are known to bind, and transport (E)-*β*-farnesene (the alarm pheromone) to ApisOR5 in *Acyrthosiphon pisum*, which made aphids escape by their walking away and falling down from the host plant.

Most chemosensory OBPs contain 120–140 amino acids and have a common folding style of six α-helical domains, among which six conserved cysteines form three interlocked disulphide bridges to stabilize the compact structure [11,12]. OBPs are water soluble and are extracellular proteins that are expressed in non-neuronal support cells of the chemosensory sensilla [13,14]. Additionally, OBPs could be categorized into pheromone binding proteins (PBPs) and general odorant binding proteins (GOBPs) in Lepidoptera [15]. OBPs were first identified and studied in *Antheraea polyphemus* [3].

Following this research, more and more OBPs have been identified and studied among insects [16,17,18]. Insect ORs encode seven transmembrane domain proteins with an inverted membrane topology (intracellular N terminus and an extracellular C terminus) when compared with vertebrate ORs [19,20]. ORs are located in the dendrite membrane of olfactory sensory neurons (OSNs), and are housed within the olfactory sensilla (mainly on the antenna) [2,21]. ORs are considered to play a central role in identifying distinct odorants and activating the OSNs [22,23]. Insect ORs form a novel heteromeric ligand-gated ion channel. These heteromers contain two subunits: a divergent conventional ligand-binding OR subunit [24,25,26,27] and a highly conserved co-receptor (Orco) subunit [28,29,30]. The insect OR protein family was first described in *Drosophila* [22].

The willow leaf beetle *Plagiodera versicolora* (Coleoptera: Chrysomelidae), is a leaf-eating forest pest, which mainly damages salicaceous trees, including willows (*Salix*) and poplars (*Populus*) [31,32]. However, there is a gap in the research regarding the identified chemosensory gene families in *P. versicolora*. In this study, we conducted a transcriptome analysis of male and female adults’ antenna in *P. versicolora*, using second-generation Illumina RNA sequencing, and then, candidate chemosensory genes including OBPs, CSPs, ORs, GRs, IRs and SNMPs were identified. The phylogenetic relationships of these genes to other insect species were analyzed. We also examined the temporal expression profiles of OBP and OR genes by performing quantitative real time PCR (RT-qPCR).

## 2. Materials and Methods

### 2.1. Insect and Tissue Collection

The adults and larvae of *P. versicolora* were collected from Sha Lake Park in Wuhan, China. The rearing conditions were 28 ± 1 °C, with 70 ± 5% RH (relative humidity) and a photoperiod of 12:12 h (light: dark). Larvae and adults were fed with the fresh leaf of willows. Around 300 male and female antennae tissues were excised from 3 day-old adults. After collection, all samples were immediately frozen in liquid nitrogen and stored at −80 °C for RNA extraction.

### 2.2. RNA Extraction, cDNA Library Construction, and Illumina Sequencing

Total RNA was extracted from collected samples using Trizol Reagent (Invitrogen, Carlsbad, CA, USA) according to protocol. The quality of RNA was checked with a NanoDrop-2000 (Thermo Scientific, Waltham, MA, USA). The Illumina sequencing of the samples was performed by Berry Genomics (Beijing, China). The cDNA library was synthetized with NEBNext^®^ Ultra mRNA Library Prep Kit for Illumina (NEB, Ipswich, MA, USA) following manufacturer’s instructions. The mRNAs were enriched from total RNA using Oligo(dT)-attached magnetic beads and mRNAs were fragmented into short sequences within an RNA fragmentation buffer.

Next, first-strand cDNA was generated with random hexamers by using mRNAs as the template. The buffer, dNTPs, RNase H, and DNA polymerase I were used to synthesize the second strand cDNA. Then, end repair was performed on these double strands of cDNA with a dA-tail was added. After the end repair and ligation of adaptors, the PCR was performed to enrich the cDNA. Finally, the cDNA library was sequenced using the Illumina Novaseq platform.

### 2.3. De Novo Assembly and Gene Annotation

After removing the raw reads containing the adaptor sequences, low-quality reads, and repeated reads, the clean reads were obtained. The transcriptome was assembled according to these clean reads by using Trinity 2.8.5 to generate a set of transcripts. These transcripts were annotated according to the following databases: NR, Swissprot, KEGG, KOG/COG, and a search conducted in the National Center for Biotechnology Information (NCBI). The open reading frame (ORFs) of each gene were predicted with an ORF finder (http://www.ncbi.nlm.nih.gov/gorf/gorf.html (accessed on 10 May 2021). Signal peptides in these sequences were predicted following the SignalP 2.0 server (http://www.cbs.dtu.dk/services/SignalP-2.0/#submission (accessed on 10 May 2021).

### 2.4. Sequence and Phylogenetic Analyses

The ORFs and amino acid sequences of chemosensory genes (File S1) were obtained and identified, after removing redundant sequences. Phylogenetic trees were constructed with amino acid sequences from *P. versicolora* and other insect species, including *Colaphellus bowringi* [33], *Ips typographus* [34], *Dendroctonus ponderosae* [34], *Dendroctonus valens* [35], *Monochamus alternatus* [36], *Dastarcus helophoroides* [36], *Anoplophora chinensis* [37] *Basilepta melanopus* [38], *Tribolium castaneum* [39], *Megacyllene caryae* [40], *Plutella xylostella* [41], *Helicoverpa armigera* [42,43,44], *Bombyx mori* [20,45,46], *Drosophila melanogaster* [47,48,49,50], *Aedes aegypti* [51], and *Anopheles gambiae* [52]. Then, we used ClustalX 1.83 to align amino acid sequences and the MEGA6 neighbor-joining method was used to construct the phylogenetic trees. Lastly, the different trees were viewed and edited with FigTree 1.4.2 software (http://tree.bio.ed.ac.uk/software/figtree/ (accessed on 15 July 2021).

### 2.5. Quantitative Real-Time PCR (RT-qPCR) Analysis

RT-qPCR was conducted to determine the expression profiles of male and female insects. The cDNAs were synthesized with HiScript^®^ III RT SuperMix for RT-qPCR (+gDNA wiper) (Vazyme, Nanjing, China), based on the manufacturer’s instructions. The specific primers were designed and are listed in Table S1. RT-qPCR was conducted on the CFX Connect Real-Time System (Bio-Rad, Hercules, CA, USA) with a ChamQTM Universal SYBR^®^ RT-qPCR Master Mix (Vazyme, Nanjing, China), following the manufacturer’s instructions. Reaction programs were set at 95 °C for 30 s, followed by 40 cycles of 95 °C for 5 s and 60 °C for 34 s. The *RPS18* gene [53] was used as a reference to normalize the relative expression levels of OR and OBP genes. For each gene, three biological replicates were conducted. Gene expression levels were analyzed using the 2^−ΔΔCT^ method [54]. The one-way analysis of variance (ANOVA) followed by the Tukey’s HSD test was used to test gene expression using SPSS 26.0 software (SPSS Inc., Chicago, IL, USA).

## 3. Results

### 3.1. Overview of the Sequence Assembly

**Figure 1:**
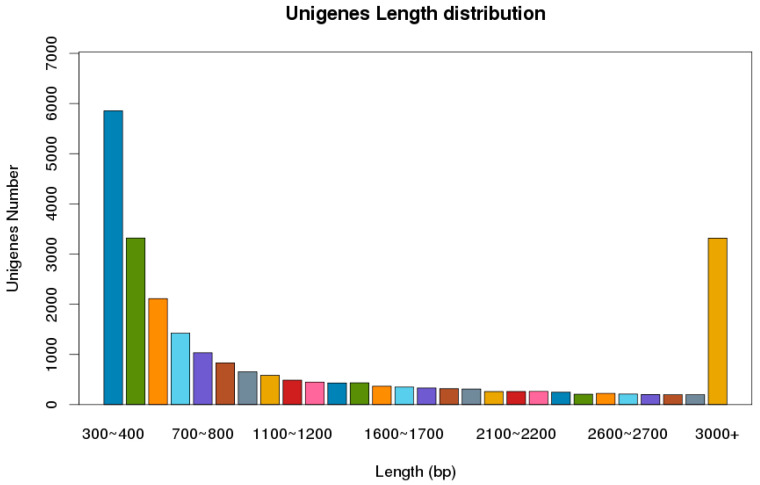
Distribution of unigene size in the *P. versicolora* transcriptome assembly.

The next-generation sequencing of the cDNA library, using the Illumina Novaseq platform, was constructed from the male and female adult antennae of *P. versicolora*. In total, 59,893,741 clean reads were obtained with a Q20 percentage of 97.83%. About 24,862 unigenes, with a total length of 34,555,981 and an N50 length of 2675 bp, were identified. Statistics showed that 63.1% of the 15,687 unigenes were greater than 500 bp in length (Figure 1). In total, 11,925 unigenes were matched to entries in the NCBI non-redundant (NR) protein database (http://www.ncbi.nlm.nih.gov/protein (accessed on 10 April 2021) by a BLASTX search.

### 3.2. Overview of Gene Ontology (GO) Annotation

**Figure 2:**
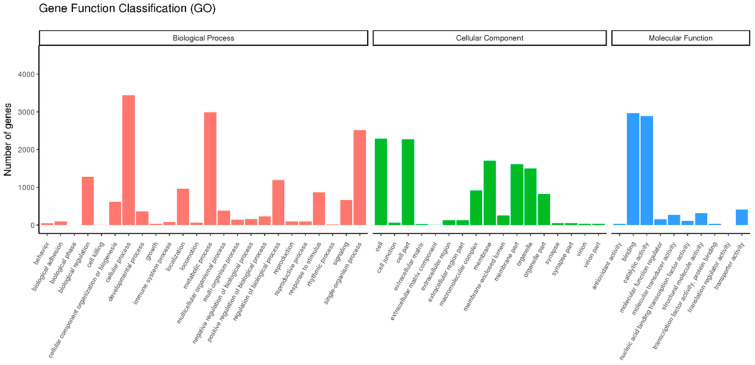
Gene ontology (GO) classification of *P. versicolora* unigenes.

The transcripts were classified into different functional categories based on their GO annotation. Overall, these unigenes could be placed into three functional categories: cellular components (11,847), biological processes (16,293), and molecular function (7150) (Figure 2). In the class of molecular function, the genes expressed in the antennae were mostly related to binding (2966), catalytic (2887), and transporter activities (406), indicating that some unigenes in these sub-categories might have a connection with chemosensory behavior in insects.

### 3.3. Identification of the Candidate Chemosensory Genes

Based on similarity analyses of the sequences tested, a total of 98 candidate chemosensory genes from the male and female antennae transcriptomes of *P. versicolora* were identified. These included 40 ORs, 7 IRs, 13 GRs, 10 CSPs, 24 OBPs, and 4 SNMPs (Tables S2 and S3). When compared with insects of Coleoptera, where the chemosensory genes had been identified by transcriptome tests, the number of chemosensory genes identified in this study was similar to those found in *C. bowringi* (104 chemosensory genes), *D. ponderosae* (111 chemosensory genes), and was higher than that of *M. alternatus* (52 chemosensory genes).

### 3.4. OBPs

**Figure 3:**
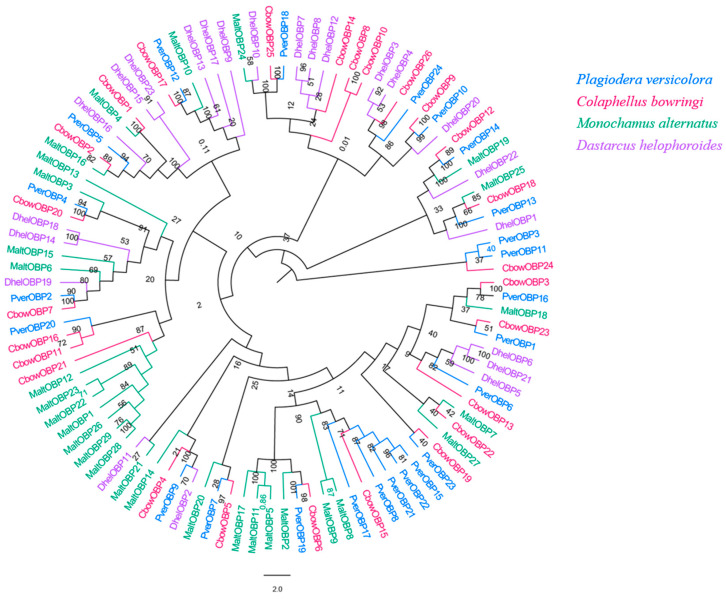
Phylogenetic tree of insect OBP. The *P. versicolora* genes are shown in blue. The tree was constructed using MEGA6 with the Neighbor-joining method.

**Figure 4:**
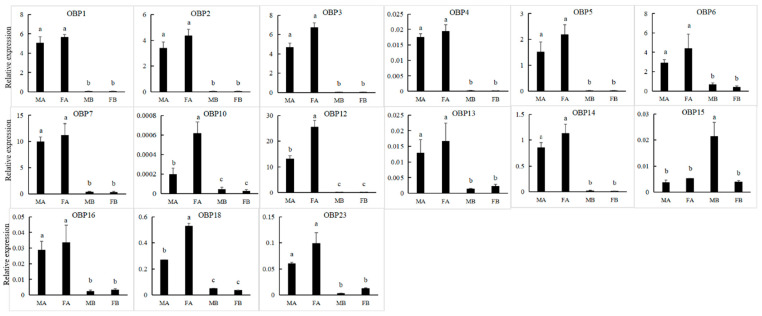
Expression levels of PverOBP genes in different tissues assessed by RT-qPCR. MA, male antennae; FA, female antennae; MB, male body (without antennae); FB, female body (without antennae). Error bars, r, are represented by the standard error of three biological replicates. Different letters (a–c) indicate significant differences (*p* < 0.05) of male or female based on one-way ANOVA.

We obtained 24 unigenes encoding candidate OBPs in *P. versicolora* (PverOBPs), which is less than that observed in *M. alternatus* (29) and *C. bowringi* (26), but more than that observed in *D. helophoroides* (23). Sequence analysis showed that 23 OBPs have complete ORFs and encoded 125 to 226 amino acids, but only three OBPs have no signal peptide sequences (Table S2). The result of the phylogenetic tree showed that PverOBP4 and PverOBP12 were clustered with the functionally characterized MaltOBP13 and MaltOBP10, respectively. In addition, several PverOBPs (OBP18, 10, 14, 16, 19, 7, 2, and 4) were clustered with CbowOBPs (OBP25, 26, 12, 3, 6, 5, 7, and 20, respectively) (Figure 3). The tissue expression profiles revealed that three PverOBPs (PverOBP10, 12 and 18) had a higher expression level in female antennae than male antennae. Among these PverOBPs, except for PverOBP15, the remaining 14 candidate genes were specifically expressed in the antennae with low or no expression level in the body (Figure 4).

### 3.5. CSPs

**Figure 5:**
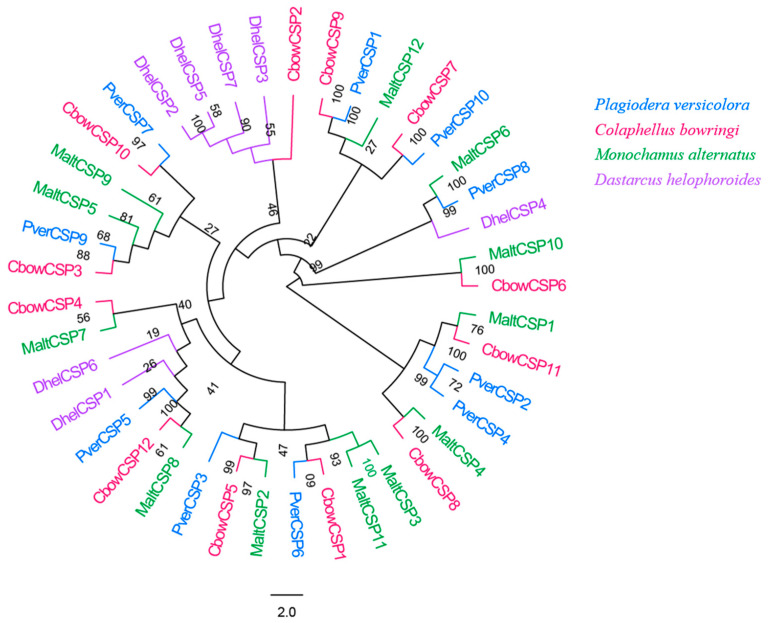
Phylogenetic tree of insect CSP. The *P. versicolora* genes are shown in blue. The tree was constructed using MEGA6 with the Neighbor-joining method.

In total, 10 different candidate unigenes encoding for CSPs were obtained in *P. versicolora* (PverCSPs), based on the transcriptomes of the antennae (Table S2). Among these CSPs, all had full-length ORFs with a predicted signal peptide. The phylogenetic trees were divided into several branches (Figure 5). The results showed that five PverCSPs (PverCSP1, 6, 7, 9 and 10) were orthologs of known CbowCSPs (CSP9, 1, 10, 3 and 7, respectively) from other insects (Figure 5).

### 3.6. ORs

**Figure 6:**
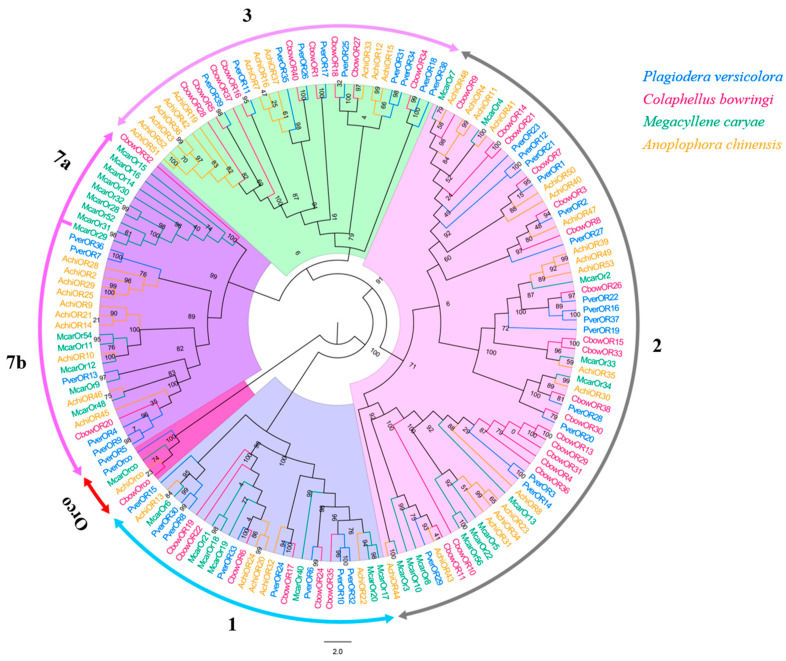
Phylogenetic tree of insect OR. The *P. versicolora* genes are shown in blue. The tree was constructed using MEGA6 with the Neighbor-joining method.

**Figure 7:**
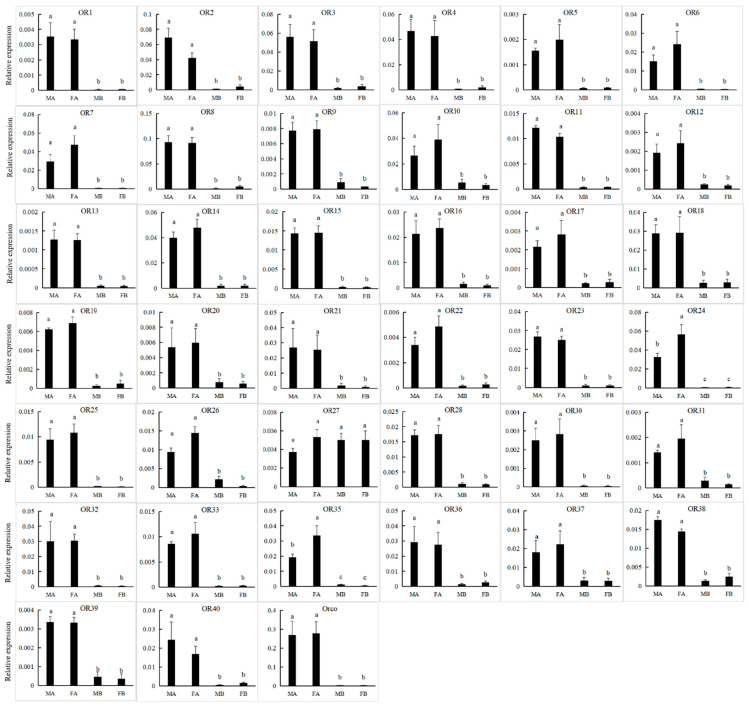
Expression levels of PverOR genes in different tissues assessed by RT-qPCR. MA, male antennae; FA, female antennae; MB, male body (without antennae); FB, female body (without antennae). Error bars, r, are represented by standard error of three biological replicates. Different letters (a–c) indicate significant differences (*p* < 0.05) of male or female based on one-way ANOVA.

Forty different unigenes for candidate ORs were identified in *P. versicolora* (PverOR), among which 35 ORs contained a complete ORFs that encoded 372 to 479 amino acids (Table S3). The phylogenetic analysis showed that a PverOR gene displayed a high homology with the conserved Orco gene family in other three insects (*C. bowringi*, *M. alternatus*, and *A. chinensis*), which was designated as PverOrco. The results show that ORs were separated into five subfamilies, those being 1–3, 7a and 7b. We found that three PverORs (PverOR6, 10 and 32) and an McarOR20 that have been functionally characterized were clustered within a subgroup. Additionally, PverOR24 was clustered with CbowOR17 and AchiOR32 in the tree (Figure 6). Among these PverORs, except for PverOR27 (which had a similar expression level between the antennae and bodies), the remaining candidate genes were specifically expressed at higher levels in the antennae than in the bodies. The results of RT-qPCR showed that PverOR24 and PverOR35 were highly expressed in female antennae (Figure 7).

### 3.7. GRs

**Figure 8:**
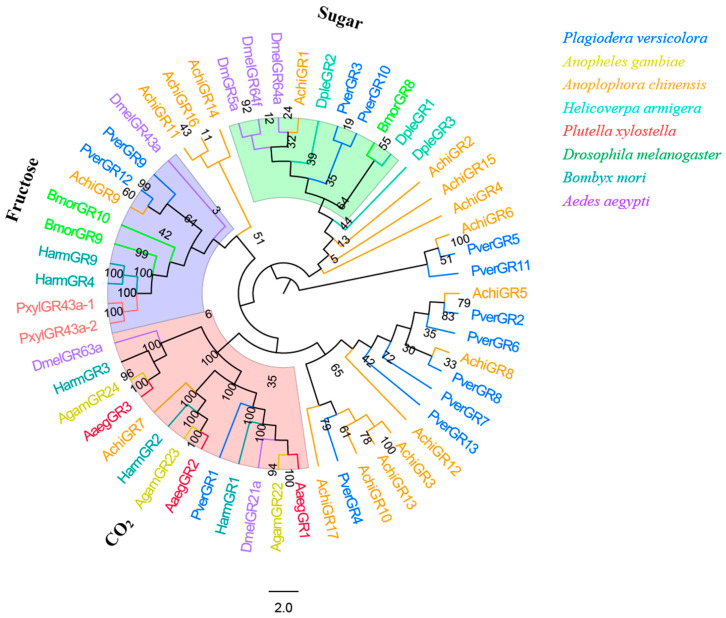
Phylogenetic tree of insect GR. The *P. versicolora* genes are shown in blue. The tree was constructed using MEGA6 with the Neighbor-joining method. The sugar subfamily does not include the fructose receptors.

Bioinformatic analysis identified 13 candidate GRs in the *P. versicolora* (PverGRs) antennal transcriptome, seven of which have full-length ORFs (Table S3). GR sequences in *P. versicolora* and other insects were used for the phylogenetic analysis. The tree showed that PverGR1 was clustered in the CO_2_ receptors subfamily, two PverGRs (GR3 and GR10) were clustered together with the sugar receptor (including trehalose, glucose, sucrose, etc., expect for fructose) subfamily, and PverGR9 and PverGR12 were clustered together with the fructose receptor subgroup (Figure 8).

### 3.8. IRs

**Figure 9:**
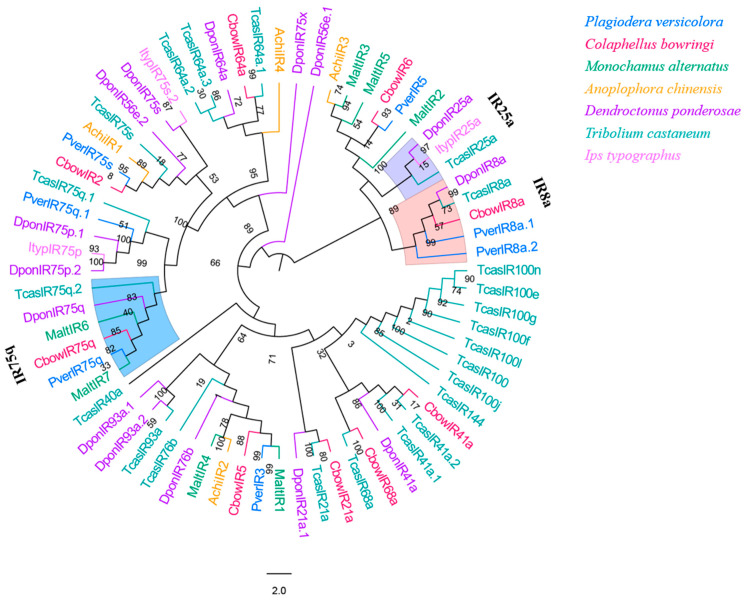
Phylogenetic tree of insect IR. The *P. versicolora* genes are shown in blue. The tree was constructed using MEGA6 with the Neighbor-joining method.

Seven IR genes were identified in *P. versicolora* from the male and female antennal transcriptomes. Only four of these IRs had a full-length ORF (PverIR2, PverIR4, PverIR5, and PverIR6) that encoded 639 to 877 amino acids (Table S3). The phylogenetic analysis of IRs from six species of Coleopterans showed that (Figure 9) these IRs can be divided into several different subfamilies. PverIR1 (named PverIR75q) clustered with CbowIR75q, DponIR75q, and TcasIR75q, suggesting it is part of the IR75q group. The results show that PverIR4 (named PverIR8a.1) and PverIR7 (named PverIR8a.2) were classified into IR8a coreceptor subgroup (Figure 9).

### 3.9. SNMPs

**Figure 10:**
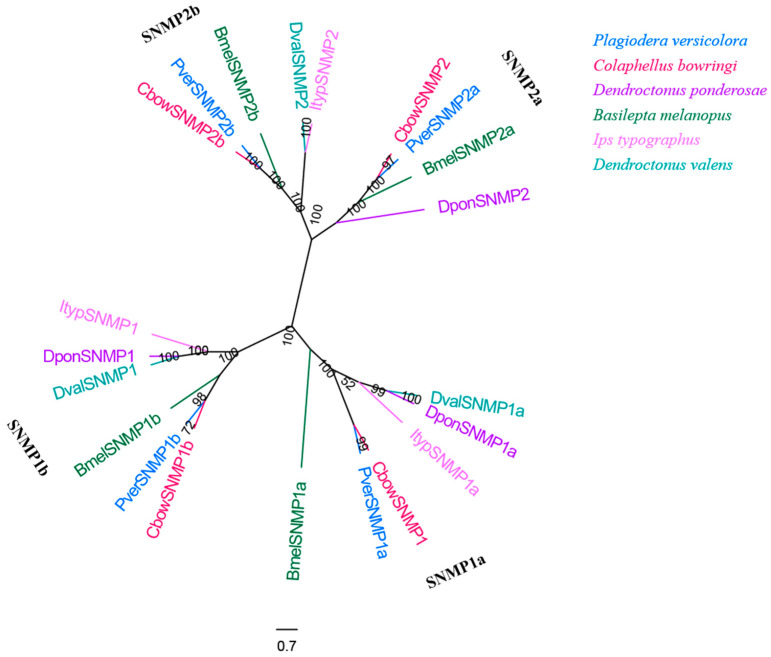
Phylogenetic tree of insect SNMP. The *P. versicolora* genes are shown in blue. The tree was constructed using MEGA6 with the Neighbor-joining method.

Four SNMP genes with complete ORFs were obtained from the male and female antennal transcriptomes of *P. versicolora* (Table S3). This number is similar to that observed in *C. bowringi* but is higher than in other insects used in the phylogenetic tree. The results also show that four PverSNMP genes were clustered into the Coleoptera SNMP1 group (SNMP1a and SNMP1b subgroup) and SNMP2 group (SNMP2a and SNMP2b subgroup) (Figure 10). Insects generally have two representatives of SNMPs (SNMP1 and SNMP2), although the copy numbers of each lineal orthologue seems to differ between species [55].

## 4. Discussion

Although Coleoptera is the largest insect order when compared to Dipterans and Lepidopterans, there has been little research into the molecular mechanism of chemoreception. In the present study, the antennal transcriptome of a Coleoptera beetle, *P. versicolora*, was sequenced and analyzed. A total of 24,862 unigenes were identified and 76.5% of them were over 300 bp in length, suggesting the high depth and quality of the transcriptome. Busco analysis was conducted to evaluate the completeness of the transcriptome (File S2). Based on the transcriptome analysis, we identified 98 chemosensory genes in *P. versicolora* (Tables S2 and S3), and the phylogenetic trees were constructed with other insect chemosensory sequences (Figure 3, Figure 5, Figure 6, Figure 8, Figure 9 and Figure 10). In addition, the spatial expression patterns of OBPs and ORs have been assessed through RT-qPCR analysis (Figure 4 and Figure 7).

OBP-binding odorant molecules consist of the first step in the olfactory process. The number of PverOBPs (24) observed is similar to those found in *C. bowringi* (26) and *D. helophoroides* (23), and less than that of *M. alternatus* (29). The phylogenetic analysis showed that PverOBP4, 6 and 12 were clustered together with functionally characterized DhelOBP13, 21 and 10, respectively. The results indicated that the three PverOBPs may have same function as in *D. helophoroides*. In the present study, the majority of PverOBPs (14 out of 15 OBPs) were highly expressed in antennae, as shown by the RT-qPCR test, which is consistent with the expression profile of genes in other insects, such as *Dioryctria abietella* [56] and *Galleria mellonella* [57]. In addition, PverOBP10, 12 and 18 exhibited highly abundant expression levels in female antennae (Figure 4), suggesting that these OBPs may play an important role in antennal recognition processes, though further verification is needed. PverOBP15 was highly expressed in the bodies when compared to other OBPs (Figure 4), which may be involved in delivering and detecting some specific semiochemicals. In line with our results, several other studies showed that OBPs are differentially expressed in the body; for example, AtumOBP5, AtumOBP17, and AtumOBP21 have a higher expression in the forelegs [58].

PverGR1 was clustered in the CO_2_ receptors subfamily, PverGR9 and PverGR12 in the fructose receptors subgroup, and PverGR3 and PverGR10 in the sugar receptors subfamily (Figure 8), indicating that these GRs might be taking part in the detection of CO_2_, sugar, and fructose [45,47,59]. Other GRs that do not belong to these three categories might be involved in other taste perception processes. Previous studies showed that an insect usually has three CO_2_ receptors (these are allocated into three different groups: one, two, and three), e.g., AaegGr1-3 were reported in *A. aegypti* [51], AgamGr22-24 in *A. gambiae* [52], and HarmGr1-3 in *H. armigera* [42]. However, only one CO_2_ receptor gene (PverGR1) was identified in *P. versicolora*, which belongs to the GR1 subfamily. Considering that gustatory sensilla are mainly distributed in the mouthparts (proboscises, labial palps), antennae, wings, legs, and ovipositor [60,61], we may identify more PverGR genes from the transcriptome of other tissues in the future.

The number of PverORs (40) is greater than that of *harmonia axyridis* (26) [62] and less than that of *Rhynchophorus palmarum* (63) [63]. In the OR phylogenetic tree, PverOR6, PverOR10, and PverOR32 were clustered into the same subgroup, with a functionally characterized pheromone receptor, McarOR20, the receptor of (2S,3R)-2,3-hexanediol and 3-hydroxyhexan-2-one in *M. caryae* [40]. The results indicate that these PverORs may be associated with the detection of the above pheromones or other active compounds. The discovery of new attractive substances would be helpful for the identification of sex pheromone compounds in *P. versicolora*. PverOR24 and PverOR35 genes were identified with significantly higher expression levels in female antennae than male antennae. Considering previous studies of the insect OR functions [64,65], female-biased PverORs may be involved in the detection of odors that play a critical role in female behavior, such as mating or oviposition. In addition, PverOR24 clusters with CbowOR17 and AchiOR32, as this might be relevant in the light of discovering pheromone receptors. The specific functions of these PverORs need to be explored in the future.

Collectively, a total of 98 chemosensory gene families, including 40 ORs, 7 IRs, 13 GRs, 10 CSPs, 24 OBPs, and 4 SNMPs, were identified through the transcriptomic analysis of *P. versicolora*. The results regarding the expression profiles of these chemosensory genes demonstrated that most PverOBPs and PverORs were highly expressed in the antennae. Besides, PverOBP10, PverOBP12, PverOBP18, PverOR24, and PverOR35 have a higher expression level in female antennae than male, indicating the genes might be crucial for regulating female-specific behaviors. In this study, we provide a comprehensive sequence resource of chemosensory receptors and insight into the *P. versicolora* chemical ecology, which lays a basis for further functional studies of the olfactory system in this pest, and sheds light on a new perspective of pheromone-based pest management in the future.

## Data Availability

Not applicable.

## Supplementary Materials

The following supporting information can be downloaded at: https://www.mdpi.com/article/10.3390/insects13010036/s1, Table S1: Primers for RT-qPCR of PverOBP and PverOR genes in *P. versicolora*. Table S2: The Blastx match of *P. versicolora* candidate CSP and OBP genes. Table S3: The Blastx match of *P. versicolora* candidate GR, IR, OR and SNMP genes. File S1: The amino acid sequences of *Plagiodera versicolora* putative chemosensory receptor genes. File S2: The busco analysis of *P. versicolora* transcriptome.
