# Supplementary material for: Identification of Chemosensory Genes Based on the Antennal Transcriptomic Analysis of Plagiodera versicolora
Source: Insects. 2021 Dec 29;13(1):36. doi: 10.3390/insects13010036 (PMC8781154; doi:10.3390/insects13010036)
Supplement: Supplementary file 1 [file insects-13-00036-s001.zip › insects-1482384-supplementary.pdf]

Supplementary data

Table S1. Primers for RT-qPCR of PverOBP and PverOR genes in *P. versicolora*.

OBP1-F	GTACTGGTTTGCTTCGCATTT	OR12-F	GCGTACAACCACGTTTCTTTG
OBP1-R	TCTGTGATTCCGCTCATTCC	OR12-R	GTCTGAAATCCAGCCTCTGTAG
OBP2-F	GGAGGAAACTGGAGCTAAAGAA	OR13-F	TGAAGAATCTGGCATTCCCTAC
OBP2-R	CAAGCCATTTGAGCCATGATAC	OR13-R	CTCCTGACTGAGCGTATGATTT
OBP3-F	CTCAAGTCAGTTGCTCCAGAA	OR14-F	GGACTATGGTTTCCAGGATCAG
OBP3-R	CAATCTCCAAAGCTCCCATACA	OR14-R	TAGCCAATCCACAGCCATAAA
OBP4-F	GCATTCACAGAAGAGGAACAAC	OR15-F	TCTACGGCATCATCGGAAAC
OBP4-R	CCACTGGATGCCCTACTTATC	OR15-R	GTATTCCTAGCCGAACCATCTG
OBP5-F	AGATATGCTGCTGAGACTTTGG	OR16-F	CGACCCATTGCTACTACCTAAAT
OBP5-R	ATGGTAGGAAGTATTTCTCTGGATAAG	OR16-R	GTGATCTGGCCACCCATAAA
OBP6-F	GAAGCAACACCACAAGGAATG	OR17-F	ATTCCGCACTCTTCACTCTTAC
OBP6-R	TGCTCCTTGAGTTTCTGGTTAT	OR17-R	CCCTACCCAGAACCAAATATCC
OBP7-F	GTTGTTCGCACTGTTTCTTGTT	OR18-F	GCACTCAACCCATACCTGAA
OBP7-R	CGGCACATTCTTCGTGTATCT	OR18-R	TAAGCGAATCCCAGCCAATAG
OBP10-F	GTACCCAATCTGGAGCAACTAA	OR19-F	GGGCTGGTTTCCATACGATT
OBP10-R	GAACTTGCCCTCGTCCATAA	OR19-R	CAAGCGAGAGTGTCCCATAAG
OBP12-F	GAGCTACCACCAGAGTTACAAG	OR20-F	ATGACAGGAGAACCAACCTTAC
OBP12-R	AGGATTCCCTTTCACGTCATAC	OR20-R	CTTCGAACGCATACGTCATTTC
OBP13-F	AGGATTTCCGACTGCTGATG	OR21-F	CTCAGCACGACCCTGTATTT
OBP13-R	TCTGAGCGAAGCTAAGACAATAC	OR21-R	CCACCGACGTCTAGCTTTATG
OBP14-R	CCGAAGAAGGAACTGACTCTATG	OR22-F	CGACTATCTGTGCCTCACTTT
OBP14-F	GCCCTCTTAATTCTGCCTGAT	OR22-R	CAGTACCCAGCATCTCGTTT
OBP15-R	GATAAGGATGCCTTGAGGAAGAT	OR23-F	GGGCATTCCTAGTTATCCTCATC
OBP15-F	ACATTTGAGCAGAGCGAGAG	OR23-R	CCGAATGCGTACAGAGTACAA
OBP16-R	CCTCAAGGAATCCAACGTAGAC	OR24-F	GCACTTCAGTTGGCAATGTTAT
OBP16-F	CCTGCACTCCGATCTTCTTATT	OR24-R	ACAGGTTCTTGACACCTCATC
OBP18-F	TGCACCATTGCATCTTCAAAC	OR25-F	GAGCGCAAGAGGATCAAGAA
OBP18-R	CATACCATCCCTTCCCTTCTTG	OR25-R	TCTCACGACCCTCACAACTA
OBP23-F	CAAAGAGCTACTCCGGAAGAAA	OR26-F	TACGTCTTGTCTTTGCCGAATA
OBP23-R	TCAACCCATACCACCGATATTC	OR26-R	GTGCTGGTACGCCGTATAAA
q18S-F	CTTCCTCGTCGGAGCATTCT	OR27-F	CGCCTACTCATCGTACATCAC
q18S-R	GTTCGCCTTAACTGCCATCAA	OR27-R	AAGTCTGGTGAACATCCACTATC
OR1-F	GGACAAGGATTTGCCGATAGT	OR28-F	GATGGACCTTATCTGCCCATAG
OR1-R	GTGTTACTGGCAACTGCATAGA	OR28-R	CTCCGACTATTGTCGCTAGTATTT
OR2-F	ATGAGCGAATGGTACGAGTATG	OR29-F	CGTCATAGCGTTGGTGTATCA
OR2-R	ATCCAGGTCCAAGTTTGTAGAG	OR29-R	AAGTTCATGCCGATGCAATTC
OR3-F	TGGACTTCTTGGTTGCCTAAA	OR30-F	CGATGATGCTGAGAAGGAGATAC

OR3-R	CAAGTCTCCTCAACGTTCTCTC	OR30-R	TGGAATCCAACAAGGGTATCAA
OR4-F	CATTCTCCTGTGCTACCTTTCT	OR31-F	GTCATGCACCAGACTCTCAA
OR4-R	CCTGCCACTTCTGATTCTATCC	OR31-R	GGAGGACTGATTTCGGCATAA
OR5-F	CTGCCTCTACTACCATGTCAAC	OR32-F	AGGAGCGTTTGGAGTTATGG
OR5-R	CGAGATCGCATCATCACTATCA	OR32-R	CTGACTCCCAAGCAGAGTAATG
OR6-F	GTCATAGTGGACAACGCATACA	OR33-F	GCGAAGACCTTTCCGTTATCT
OR6-R	GACATCTCATCAGGACGAACAG	OR33-R	GAACTCTGACCATTTGCTTTGG
OR7-F	GTCACCCAAACTTTCTTCATCAC	OR35-F	CATGTCCTACAACTTGCTGAAAC
OR7-R	GCTTGCTCATCACCGATACT	OR35-R	GTGTTCATTGCAGCCCAATAC
OR8-F	GCAACTTGGTGGTTGGATTG	OR36-F	TTCCCTTGCCATAACCTTCTAC
OR8-R	GCTGCCTCGCTCATAGATAAA	OR36-R	TGTGTTGGTACCACCGTATTT
OR9-F	CCAACTGGTATGAACGATCTCC	OR37-F	CGGGCTCCAGTGTGATTATT
OR9-R	GCACAACTATCCCTCCACTTT	OR37-R	CTACGAACCTCTTCATCCTCATC
OR10-F	AGGAGCGTTTGGAGTTATGG	OR38-F	CAGGGACTTGGAAGGCAAATA
OR10-R	CTGACTCCCAAGCAGAGTAATG	OR38-R	AGTCATGTAACCACACGAACAT
OR11-F	GGCCTATATGCTCGTCATAGTG	OR39-F	TTTCTGAGCCTCTTCTCCAATC
OR11-R	AAGAGTGTCGCCGAATAAGAG	OR39-R	GATACCTCTGGACTCTGGTAGT
Orco-F	CTGTAGCCTGGACATCCATAAC	Orco-R	GGAGCCTGGGAATTTCTTCTAT

Table S2. The Blastx match of *P. versicolora* candidate CSP and OBP genes

Name	ORF (aa)	SP	complete	Blast best hit ACC. NO. Gene Species		Evalue	Identity (%)
CSP1	150	1-20	Y	ALR72523.1|	chemosensory protein 9 [Colaphellus bowringi]	8e-58	64.8
CSP2	118	1-25	Y	ALR72525.1|	chemosensory protein 11 [Colaphellus bowringi]	9e-53	73.4
CSP3	128	1-17	Y	QFO46789.1|	chemosensory protein [Cylas formicarius]	5e-56	68.8
CSP4	114	1-25	Y	AIX97041.1|	chemosensory protein 1 [Monochamus alternatus]	6e-20	72.1
CSP5	134	1-16	Y	ALR72526.1|	chemosensory protein 12 [Colaphellus bowringi]	1e-68	80.7
CSP6	126	1-18	Y	ALR72515.1|	chemosensory protein 1 [Colaphellus bowringi]	4e-52	71.2
CSP7	131	1-15	Y	ALR72524.1|	chemosensory protein 10 [Colaphellus bowringi]	5e-43	53.9
CSP8	213	1-18	Y	AIX97046.1|	chemosensory protein 6 [Monochamus alternatus]	5e-64	62.8
CSP9	131	1-18	Y	ALR72517.1|	chemosensory protein 3 [Colaphellus bowringi]	5e-48	64.9
CSP10	127	1-18	Y	ALR72521.1|	chemosensory protein 7 [Colaphellus bowringi]	3e-66	79.5
OBP1	131	1-19	Y	AIX97052.1|	odorant-binding protein 6 [Dastarcus helophoroides]	4e-25	40.3
OBP2	133	1-18	Y	ALR72495.1|	odorant binding protein 7 [Colaphellus bowringi]	4e-67	75.2
OBP3	125	N	Y	ALR72489.1|	odorant binding protein 1 [Colaphellus bowringi]	7e-21	52.8
OBP4	132	1-17	Y	ALR72508.1|	odorant binding protein 20 [Colaphellus bowringi]	9e-56	60.6
OBP5	140	1-19	Y	ALR72490.1|	odorant binding protein 2 [Colaphellus bowringi]	1e-57	59.7
OBP6	135	1-18	Y	AWK23450.1|	odorant-binding protein 13 [Chrysomela populi]	3e-81	94.8
OBP7	131	1-17	Y	AXO78397.1|	odorant binding protein 19 [Xylotrechus quadripes]	3e-21	43.6
OBP8	142	1-19	Y	APC94279.1|	odorant-binding protein 4 [Pyrrhalta aenescens]	4e-21	46.3
OBP9	111	N	N	ALR72492.1|	odorant binding protein 4 [Colaphellus bowringi]	4e-33	58.2
OBP10	155	1-20	Y	ALR72497.1|	odorant binding protein 9 [Colaphellus bowringi]	2e-39	65
OBP11	170	N	Y	ALR72505.1|	odorant binding protein 17 [Colaphellus bowringi]	2e-13	85
OBP12	136	1-18	Y	ALR72505.1|	odorant binding protein 17 [Colaphellus bowringi]	8e-70	75
OBP13	183	1-23	Y	AUF72969.1|	odorant-binding protein [Anoplophora chinensis]	3e-84	70
OBP14	241	1-18	Y	ALR72500.1|	odorant binding protein 12 [Colaphellus bowringi]	1e-61	47.2
OBP15	129	1-19	Y	AQY18986.1|	odorant-binding protein [Galeruca daurica]	5e-26	40.6
OBP16	134	1-17	Y	AXO78395.1|	odorant binding protein 17 [Xylotrechus quadripes]	2e-41	47.7
OBP17	137	1-21	Y	ALR72503.1|	odorant binding protein 15 [Colaphellus bowringi]	2e-16	40.4
OBP18	266	1-20	Y	ALR72513.1|	odorant binding protein 25 [Colaphellus bowringi]	1e-85	59.6
OBP19	135	1-19	Y	ALR72494.1|	odorant binding protein [Colaphellus bowringi]	4e-46	65.2
OBP20	152	1-23	Y	ALR72504.1|	odorant binding protein 16 [Colaphellus bowringi]	1e-24	36.7
OBP21	138	1-19	Y	AQY18990.1|	odorant-binding protein [Galeruca daurica]	1e-21	40.8
OBP22	133	1-19	Y	ALR72503.1|	odorant binding protein 15 [Colaphellus bowringi]	3e-25	55.2
OBP23	159	N	Y	APC94275.1|	odorant-binding protein 27 [Pyrrhalta aenescens]	5e-26	42.9
OBP24	148	1-16	Y	AWT23276.1|	OBP5 [Hycleus cichorii]	9e-30	37.6

Table S3. The Blastx match of *P. versicolora* candidate GR, IR, OR and SNMP genes

Name	ORF (aa)	complete	Blast best hit ACC. NO. Gene Species		Evalue	Identity (%)
GR1	436	Y	QBB73006.1|	gustatory receptor [Protaetia brevitarsis]	0	73.1
GR2	103	N	APC94248.1|	gustatory receptor 3 [Pyrrhalta maculicollis]	4e-30	59.4
GR3	173	N	AUF73052.1|	gustatory receptor [Anoplophora chinensis]	6e-40	40.8
GR4	381	Y	EFA02932.2|	gustatory receptor 98 [Tribolium castaneum]	6e-167	60.8
GR5	384	Y	AVN97871.1|	gustatory receptor 6 [Anoplophora chinensis]	3e-13	26.7
GR6	100	N	APC94248.1|	gustatory receptor 3 [Pyrrhalta maculicollis]	3e-27	57.3
GR7	390	Y	APC94333.1|	gustatory receptor 3 [Pyrrhalta aenescens]	2e-28	28.7
GR8	382	Y	ALR72586.1|	putative gustatory receptor GR9 [Colaphellus bowringi]	2e-14	55.1
GR9	426	Y	AVN97874.1|	gustatory receptor 9 [Anoplophora chinensis]	4e-80	38.2
GR10	480	Y	APC94346.1|	gustatory receptor 8 [Pyrrhalta aenescens]	6e-76	51.4
GR11	131	N	AWK23449.1|	gustatory receptor 1 [Chrysomela populi]	3e-67	79.4
GR12	187	N	AVN97874.1|	gustatory receptor 9 [Anoplophora chinensis]	1e-26	36.4
GR13	127	N	AVN97873.1|	gustatory receptor 8 [Anoplophora chinensis]	6e-14	43
IR1	462	N	ALR72537.1|	ionotropic receptor 75q [Colaphellus bowringi]	8e-177	58.4
IR2	639	Y	AKC58589.1|	ionotropic receptor 75q [Anomala corpulenta]	2e-95	33.8
IR3	589	N	ALR72540.1|	ionotropic receptor IR5 [Colaphellus bowringi]	0.0	61.3
IR4	877	Y	ALR72538.1|	ionotropic receptor 8a [Colaphellus bowringi]	0.0	65.9
IR5	847	Y	ALR72535.1|	ionotropic receptor IR6 [Colaphellus bowringi]	0.0	83.5
IR6	639	Y	ALR72541.1|	ionotropic receptor IR2 [Colaphellus bowringi]	0.0	65.9
IR7	98	N	QGW45446.1|	ionotropic receptor 8a [Bradysia odoriphaga]	2e-09	85.7
OR1	381	Y	ALR72552.1|	odorant receptor OR7 [Colaphellus bowringi]	2e-93	47.5
OR2	395	Y	ALR72548.1|	odorant receptor OR3 [Colaphellus bowringi]	2e-145	57.8
OR3	423	Y	ALR72579.1|	odorant receptor OR36 [Colaphellus bowringi]	7e-75	48.3
OR4	384	Y	ALR72565.1|	odorant receptor OR20 [Colaphellus bowringi]	1e-60	43.3
OR5	237	N	ALR72565.1|	odorant receptor OR20 [Colaphellus bowringi]	4e-57	43.2
OR6	399	Y	ALR72568.1|	odorant receptor OR24 [Colaphellus bowringi]	0.0	67.5
OR7	403	Y	QXE93269.1|	odorant receptor 43 [Eucryptorrhynchus brandti]	1e-56	31.2
OR8	392	Y	QXE93264.1|	odorant receptor 38 [Eucryptorrhynchus brandti]	1e-89	41.4
OR9	383	Y	ALR72565.1|	odorant receptor OR20 [Colaphellus bowringi]	4e-57	40.6
OR10	383	Y	ALR72565.1|	odorant receptor OR20 [Colaphellus bowringi]	7e-47	40
OR11	302	N	ALR72561.1|	odorant receptor OR16 [Colaphellus bowringi]	5e-34	51.2
OR12	386	Y	AJO62235.1|	olfactory receptor OR16 [Tenebrio molitor]	1e-24	25.6
OR13	393	Y	QXE93225.1|	odorant receptor 48 [Eucryptorrhynchus scrobiculatus]	1e-69	1e-37.1
OR14	421	Y	ALR72579.1|	odorant receptor OR36 [Colaphellus bowringi]	2e-114	46.9
OR15	389	Y	EEZ97750.2|	odorant receptor 102 [Tribolium castaneum]	9e-104	47.3
OR16	413	Y	ALR72569.1|	odorant receptor OR26 [Colaphellus bowringi]	3e-100	43.7
OR17	365	Y	ALR72546.1|	odorant receptor OR1 [Colaphellus bowringi]	4e-89	43.4
OR18	451	Y	ALR72577.1|	odorant receptor OR34 [Colaphellus bowringi]	3e-157	51.3
OR19	401	Y	AUF73016.1|	odorant receptor [Anoplophora chinensis]	2e-102	43.5

OR20	402	Y	APC94308.1| odorant receptor 3 [Pyrrhalta aenescens]	2e-82	39.3
OR21	376	Y	AVN97853.1| odorant receptor 41 [Anoplophora chinensis]	1e-32	26.9
OR22	403	Y	ALR72569.1| odorant receptor OR26 [Colaphellus bowringi]	6e-164	61.1
OR23	374	Y	AJO62235.1| olfactory receptor OR16 [Tenebrio molitor]	3e-37	31.1
OR24	386	Y	ALR72562.1| odorant receptor OR17 [Colaphellus bowringi]	0.0	62.5
OR25	418	Y	APC94306.1| odorant receptor 2 [Pyrrhalta aenescens]	6e-61	33.6
OR26	368	Y	ALR72583.1| odorant receptor OR40 [Colaphellus bowringi]	0.0	70.0
OR27	188	N	AUF73039.1| odorant receptor [Anoplophora chinensis]	3e-21	34.8
OR28	420	Y	APC94308.1| odorant receptor 3 [Pyrrhalta aenescens]	2e-153	54.0
OR29	417	Y	APC94330.1| odorant receptor 26 [Pyrrhalta aenescens]	0.0	61.3
OR30	392	Y	QXE93264.1| odorant receptor 38 [Eucryptorrhynchus brandti]	3e-72	37.1
OR31	372	Y	EFA02957.1| odorant receptor 314 [Tribolium castaneum]	4e-10	27.0
OR32	388	Y	ALR72578.1| odorant receptor OR35 [Colaphellus bowringi]	2e-149	57.9
OR33	386	N	ALR72551.1| odorant receptor OR6 [Colaphellus bowringi]	6e-172	63.7
OR34	129	N	EFA02951.1| odorant receptor 197 [Tribolium castaneum]	4e-10	28.8
OR35	384	Y	ALR72564.1| odorant receptor OR19 [Colaphellus bowringi]	1e-125	46
OR36	384	Y	QXE93269.1| odorant receptor 43 [Eucryptorrhynchus brandti]	1e-51	31.4
OR37	227	N	ALR72569.1| odorant receptor OR26 [Colaphellus bowringi]	2e-61	45
OR38	426	Y	QXE93221.1| odorant receptor 41 [Eucryptorrhynchus scrobiculatus]	1e-73	31.3
OR39	373	Y	AVN97831.1| odorant receptor 19 [Anoplophora chinensis]	2e-58	32.4
Orco	479	Y	ALR72547.1| odorant receptor ORco [Colaphellus bowringi]	0	91.4
SNMP1a	515	Y	ALR72542.1| sensory neuron membrane protein SNMP1a [Colaphellus bowringi]	0	66.0
SNMP1b	534	Y	ALR72543.1| sensory neuron membrane protein SNMP1b [Colaphellus bowringi]	0	54.9
SNMP2a	510	Y	ALR72544.1| sensory neuron membrane protein SNMP2 [Colaphellus bowringi]	0	57.4
SNMP2b	506	Y	ALR72545.1| sensory neuron membrane protein SNMP3 [Colaphellus bowringi]	0	63.6

File S1. The amino acid sequences of *Plagiodera versicolora* putative chemosensory receptor genes.

OBP

>PverOBP1

MKIIVLVCFAFAFFENGKCFSDEEKTMIMNIHTECVGMSGITDAMIEQARTGDFPPDSEFKEYLLCFAKKAG
VMNESGELQTGKITKMVQMHIADAAKADKMVETCLVNKETPQDTIFETAKCMFEVYRFF

>PverOBP2

MEIWMVVGLLLSTDAVMGLSEEMQELADMLHATCVEETGAKEEDIVNARKGDFADNENFKCYTKCIMA
QMACIDDDGIIDEEATIAVLPEEYRSKSAPVIRKCGTVKGASPCENAWLTNKCYQHEAGNEYFLV

>PverOBP3

MLCIMNTYKLVRFNNNFTCTTSIFNSFQFSKEGSFDWQTGVQTLKSVAPEKIAGPGVVSIQNCKDAKKATD
KCMGALEIAKCIYNDNPQVSNDSPFYSLSEWKYDFKVVRKNSLDIGSTFSIDNS

>PverOBP4

MFSFIVVGLCIICSISAFTEEEQQMMEALHAECVSQTGCPEDLISRASSGDFPEDEKLKCYMKCIFNELGVI
DDDGKIDSSGLVAMFPEDIQAIAKPIFAKCGTVAGTDLCDSIYQTNKCYYGENPGAYFLP

>PverOBP5

MLKGIVIVFLSSVILDVTGMNEKQMQAAVKVVRNVCMPKTKATADDIDKMHRGDWDIEYSAKCYMSCS
LNMYKLMHANNTFNYESAQKQLKTLPDTYLESTKMSIDNCRYAAETLDDKCIAAYEVAKCVYLTYPEKY
FLP

>PverOBP6

MKFAVVVVLAVAVCFVQGDLSPEQVEKVKQHHKECMQSSGITPELLAKTKKGEFPDNQKLKEHMYCFAK
KAELMDAQGKIKKDVLLAKATTALGDRALAQKLIDECAVQKKDGPETAFQCAKCYYEKTPSHLSLV

>PverOBP7

MGKVLFALFLVFTVAFCKPLSDEVKAEILKIHEECAVTSGVNPREILDKVFGEGGDEDQIKTHVFCIGQKLK
VIDDDNKIDRETLKTHLSEIITEDGEVEEIVTKCAVEKDDAKETAYYLTKCIHESMQKE

>PverOBP8

MRPIVLIASILVLVAVVAADPKKVNPKVEEFKRIHDECQADPATKVDHETMEKFYKGESIDEVKFGKHILC
MNIKMGVQNEHGDIDKEALKKGLGEHNEKAQEIADECGEKKGDTAAEAALALAKCYRKHRPAPPRDHA
HRH

>PverOBP9

MKEREKIGLECLKIVNIKKEVIEEIVKTMIFPKENQKYKDFLACSYKKQGFQSQEGHILWDNITEFISRFYK
NQDLKILDKCKSTSGKNHGEMAFNAMECIMNELLYMDDN

>PverOBP10

MKSTYFTLLLATSLFSATLGARRGRTGLLDPSNHKKVLNDCRTQSGATNSDMEAIKMKKLPDTKTGRCLV
QCIFNNAKIMDEGKFNKNGMVIAFTPALKGDLTKLGKLKQLSEVCDKEIGPAVSKDCEGPKKIVECVAKH
GSAYGFSYDNTNVNL

>PverOBP11

MLISKFKVIPYNKSRFVFHFRSSTNSEINAGSDYSYKLYSIESDFRYRKLEIPKSKHVSRIQLMKNTSYGAFP
NRKDFSLALNSISHQRLNRNSNFILEHHSENNKIHTSCRRDLSNDHIHILILVNIFTSGSASYMQKLKFDAFS
HKFAPSSIFLHLLMAGSRRSLIVG

>PverOBP12

MTLKSFLYFTVLLGLGSCIELPPELQEFVEDLHKICVSKSGITESDYAAYDVKGNPHDTKLQCYMKCLMM
EAKWMNSAGAIQYDFIIDTAHPTIKDLLEPAINKCRKIDEGANLCEKASNFNFCMYEADPENWYLI

>PverOBP13

MSTVRHCLLYGVCFLVFNVRIEAAEIHKNYTNKCDIPPTAPKKIEAVINQCQDEIKLAILSEALESLNVNEHT

HSRAKRAAFSDDERRIAGCLLQCVYKKMNAINDKGFPTADGLVSLYTDGISQKDYILATVDAVKYCLSFA
QKKFRVTPNSIEVHGMSCDIAYDVFDCVSDEIANYCGQSP
>PverOBP14
MRGLILIFVSCYVVHSKAIECGIDKSNRDEIKQALAMCVKNNATLNKIWEMTSSAQTTPSSTEEGTDSMEE
NDKSVPSIPTRNIAKNQRSGKSGRIKRAKSMKSFNTQKFSTTTMRSSDDSNGMKNSDEEENNESNDVSED
KKDSSQDSTDKCIIHCVLEKMSLTDDNGLPDHSKILEELLKNTPKRELKNFLQDSTDECFQEVDEANESDS
CEYSNKLIFCLAEKGRSNCADWPAGSLPF
>PverOBP15
MKPIALVAFILVLVAMIAAEDIHSRMKNIHEECQADPATKIDHGVMEKFFEGEKVDQDQFARHSLCMNKKI
GLQKENGDVDKDALRKIFEHNDKLDEAVEECGQGKGTAEETALALLKCIKKYRPSRTP
>PverOBP16
MNSALVLVICLIGIVKAGMLTDEQKEKILKYGQECLKESNVDFDVVMDAAKGKYADDPNLKKQILCFNK
KIGVQDSDGKLVMDTVKARLMGITNDPKKTEDIIKECVIEKSTPEDTAFETAKCLHRLAPDEKIV
>PverOBP17
MKIFLVTICVVSFVVMALVSGKDLRIKTYFHECQSDPKTYVDEELLQKGLKGELADETPVGPHAFCMNLK
RGFQVPNGDVDVVALRTYLEQSESRNDTLIDRAIKECGQRNGSTAQQAALALLDCIHRIIPHKDENH
>PverOBP18
MMKTLTQLVLVATLTTVIVAYDFQNAEFNQILAEDLEDVYTNTFYHPRVRREETTSEEDKCRPRRGKPLCC
GEELFRKPNDDDKDIKRACFKEITGKDKPEGRPDKHHGGPFDPFNCEKVEQFRKDMLCIEQCVGQKRNII
DAEGNLKQEDFQKYVQNQLAKDPALASITDKVVAGCLEEVKNATSLPQKDDSSCKPVGIALHHCIFKQVQ
LNCPEEQIKDKTACARFQERLKKGRDGMFGGPPGPGGPGGPPGPPGPPGPEGDDE
>PverOBP19
MKSTLVVFSCIVVAVLANSLPESERLKLARVHAECQANPKTRCDENLLRNLGANANNAQVGIHMLCMSV
KEGLQRPNGDLDRNFIKSKIDLVSDDKSKVDHYLQTCAVKKQTPEKTAVDLVLCFVQNGIPYYYRL
>PverOBP20
MYYNIYSIVFAIILITSMKYSSAELRHEDLGEGLLKLAELLHNTCINKTGTDDAAIDNLRNGNFVDDDKIKK
YVACVWMDAQLVRPDGSMNEQLMKDLCPPKIRENGPRIVMQCWKQVKKVIPIEEKIYEMLECYYKIDPE
VSTKMSECLSI
>PverOBP21
MKSIALIVFILAFTAMIDADDIQDEREKARKVHGECQADPATKIDEDAMDRFFRDEAVNEAQLAKHSLCIH
KKMGLQNENGDIDKDLLRKLIRHDSNVDEIVAECGQRKGSAEETTLALLKCLKKYRHREDEHHHHGH
>PverOBP22
MKSIALIAFILALGAMIAADDIQERVEKMKKIHGECQADPATKVDEEDMEKFFRSETIDEAQFAKHTLCMN
KKLGLQKENGDVDKDNLRKSAERSDKVDEIVEECGQRKGTAEETALAVLKCIRKYLPKQEHH
>PverOBP23
MLWIEWMDHRILFEMISRTPNLMKSAEKLSEYSREIFLNVLQIFPGGQLGTVVRIHRECQSRTGVSDATAL
GATSGRFPNDPAFRSHLLCMNQRMGFQDNNGNINRNAISRSLRTAAPNANIEGVLQNCAIQRATPEETAFV
MDQCFFTSLRSNIGGMG
>PverOBP24
MQRLIVFAALTIAVQSVDQSFINEFKEKATEIGIKCVEQTGASNDDIASIMDQKVPSSKEGKCMISCFHKAV
GVQNDDGSLNPNGMKVFMEKLKSNDEDMYNKFRQLLDECLIPGNVLPDHCDTSAKVATCAIEVAKKVG
LSSSFMKL

CSP

>PverCSP1
MNYYCFFIFSIIVIPQVIDGGDVGVNFLRRVERAVAEKYSTKYDNFDVLSVFASSRLVKRYGDCLMARGSC
TPEGKFLKDVIPDAIATKCSKCNAKQKKLAGVMLQQLLLNYRTLFVELCDKYDPSGEARKMYGIDTDTN
GTDYENYDEA
>PverCSP2
MQVQNSPVLVMANIVILSIVLTATARPASLTERPTVSDDALDSTLKDKRYLMRQLKCALGEAPCDPVGRRL
KSLAPLVLQGSCSQCSPQEQRQIRKVLGYMQVNFPKEWNKILKQYSG
>PverCSP3
MGVCTIFLICLVGLVMAKPDEKKYTTKYDNLDIDTILKSERLLKNYVHCLLDKGKCTPDGAELKEHLPDA
LLTDCSKCSEFQKKNSKKIIRYLIDNKPDWYKELEVKYDPEGTYKAKYDEEIKDKAAE
>PverCSP4
MQVQNSPVLVMANIVILSIVLTATARPASLTERPTVSDDALDSTLKDKRYLMRQLKCALGEAPCDPVGRRL
KKLKKYLFDLRLLDTAYVFLALEIWKPAKNKMMNHDICSQSVV
>PverCSP5
MFSLFILSCFVIGSLSTVTEKTKYTTKYDNINVEDIVKNDRLLKNYVDCLLDRGKCTPDGLELKKNMPDAI
ETDCSKCSEKQKEGSEFMMRYLIDNKPAYWDPLQEKYDPSGSYKKRYLDAKKTEVNIQPIVKS
>PverCSP6
MHLFSIFIFVFLVVSAIGEKYTTKYDNIDLDEILKSERLLKNYIDCLMDKGNCTPDGKELKETLPDAMRTEC
SKCSDKQKEGSKKVLKFLLKNKRATFDELASKYDPEGYYVKRYKDELAKEGIII
>PverCSP7
MKTILLLAFIGICAAKYTTMYDNVNIEQILKTQRLLSRYVDCLKWVPNACNKEGMKLREVLPDALLHGC
KECSEQQKKNAKRVANYMIECHPKWWSELAQIYDPRGTYQTTHQAELKAQDGSKSKNIVCYH
>PverCSP8
MLPLLPIFVCGLSGLVVAGPLQYYATKYDHIDVETILNNRRMVNYYAACLLSKGPCPPEGVEFKRILPEALR
TNCLRCTEKQKTVTLRTIKRLKKEYPKIWTQLQDEWDPNGTYVTKFENTFINSYKEPTSSIPVIIKDRFGED
NEEKIIENTVGSISSSTTSTTTIKTTKPTTYTTTTKAISKTSTIKKSSSTTQKNHQETHHSFRINTNHF
>PverCSP9
MKCIIVFCFVVVLASVIARPDDSKYTSKYDNVDLDAIIHNDRLLRNYVDCLLGKKKCTKDGEELKRILPD
ALKTKCSKCSEFQKNSAKKMVNHLIKNKRAWWDELEAVYDSNGIYRKEYEEEAKKEGIDLN
>PverCSP10
MKSVSALIFLSVLAIVASQNSYTSKYDNVDVDKILKNDRVLSNYIKCLLEEGPCTPEGRELKKTLPDALAN
GCEKCNPKQKDTSEKVMKHLAAKRARDWERLVKKYDPQGLYKKRYEQQLSKSNPAN

OR
>PverOR1
MSSTDAMYQAISIPKYFLTLTGQWPVTSPTIFHSIMSQVSFAFAFFFSFSLAFECSKHLDDYQVLSEILSIGIPP
TALVFKLAVFKMKSGYLLEVIEHLDWEEFNSHSAEANGRLWEAVRFTRYLGSSYQVTAFMVSFLYSIMPL
MSDKDLPIVYSYEADWMFFVVYVFQVVGLFYAVASNTCIDVLAVSLMGIASAQIDILNFNISNLKLVSIDSE
NDGIEGNNSEFNACVKHHNRIIRFVESLDNVFSLTILIQYMTSAACICNVGFQLVHTPSDDIIQMVKMFVFFI
AMMCQLFLYCWYGNEITVKSTAIRDACYESQWYDSELKTRRSLLTIMERTKRPLFLTSGQFYVVSLQSFTS
VIQTSYSYFALMQTLMEEEE
>PverOR2
MTSLVKQSFGRNLTVLKLVGLYPYDRYPKIFKIYAYFLYFLCTVPVPLLATVNLIVKRETDLAKISDTLFVIL
EVGICILKFLPFKNKPEAIKRTIYIVDDDEFNRATKDQFHVVEEAAKSCRRMFDVFLILCLGSLFTWPIKVLF

YEQRKFPIDVWLPFEPFEDIRVYLGLFLYIFVATGNAPIGNAAVDTLIPGLMCHAATQLKIIKDKLENLGQRV
EKQISEKYADISIEERIELKNALIYREISENVEHYNVIFRFMNEIQDTYGFVILTQFVVSIMVICMACLQLSITE
PLTPLFFAMLIYIVAILIEVLFYCYYGTILYEESNSMVQAIYMSEWYEYDEKCKRALITLMERTKRPMIMTT
GKLVDLSLQTWTWIIRRSYSLLAVLNSYN
>PverOR3
MSVARKDFGYPEDVFTVTVLVQKLAGIWIPGSDNNILFRIFYMTFVALLYGVGILFLICEIVIFYKSLTDIEKV
MSNISMMLTHLVGAFKFCVVAFGIEKIKKIMDTLQDEKYQYVAMGDFHPGEMMNKQKTRNSILSLLFFSL
YFMAGTSAHISTIFSLNIGMSGDTFERINTTCYDIMPYVFHIPFPTDKRWECNIAAAFMNIGLQLFAGVIASN
DAIFVGLLGCLKTQMIIVGHVFKTMRERTLRRLDFPEKYALMHDSDNPALEKELYSQLNKAIEHLMVLLR
ARDDIEATFTYVTLSQSIASLFIIASCLFVASTVPIGSPNFFSQLEYFVCVLVQFSLICWCGNEITIASETITTAL
YESDWFSSTARYKTSMIITMTRMQRPVYLTIGKFTPLTLTALITVFRGSFSYFTVFKSIQ
>PverOR4
MELRRILKYTRYVMLVAGALKIPNHGYLSFSYNAWAYISQAYYVYFCVSFALGLKTALDKEENVDFNKQT
ILLCYLSLWKMLLSYSTRIRNSMEALQGIESEVAGDDPKVIEIYNRASRYNHKMFFFLSFVYGLTSYFSLSE
SISNWVLMHRGNATECSLKVAGWFPFDTDRFFVEAYFFQAVAALFIGFYSLTFDSLFVSLMNFPSAIFNVLG
YKLENLDLYSTERNVSSNICLKDIIRQHQDIIQYIEDLNNSLKWFFFGDFLIKSYHISLALNNVVHTSKETFG
LYTFILTFLFTQVFCLYYHANEIILKSTSLSKRIFESNWYEQTPEVKRSLMIVMMRSRKPLNLTIGFLHVIDDE
LIVKIMKAVYTFVLFQLDLYGTQ
>PverOR5
MRTENTAQSDSLLFLSWFPFDSDKYFDVAYFYQVSIGFFIVLYSSMSDSLFISLVNFATARLVILGYKFEDLE
TFSLREKRPVCEFLGDLIMEHKDIIRYVEDLNAMLRWYFFGDFLVGSYHISLAIMNVRHHISEGSIFFHSFYL
MYILAQVCCLYYHVNELILESTNLSRKIYESKWYDQTLRVKRSLLIVMMRSRKPLILTNGNFGIIDNNLFVK
ILKAAYTFLLYQLLDILRR
>PverOR6
MNDIEKEENAVKPRDIICMPTSIKVFRLYGMFPSANRLLNPGTKFYIKFAMIALYSSLVLVGCTMHLVKSIK
DNTYNHVELDITYIVSMCAGYGLICSYITKVKFAVELYLFLSDFSEFKKPIDFEETNKKYNRYSIYHYCYLE
SIVIFILLGSNMFKGEQCRRENEANNLNEVCGLFTYTWMPFNIDFIPVKQMYLFEQLFGAHYVYMVAGLA
AWMVLESVEHIASRIRHVTYLFNESMNERDPKERRRKFNFAVRYHVAVLKLEDKLNKTFSVFMFTHMVM
TGAIMGYGVYAYIKGRNLSSFLLAIGWLVGLLMDCHSGQRIQDESDSIAMGLYQANWDQCDSELKKDILF
VLMRCQKQMILKAASFGVMDHPMFLAVLKASYSYITLMNQNEKRDH
>PverOR7
MKNFKDFDIKHFKFSMYLMIVSGTWRLELVTESKIVKTCYRMYTILIQSVFVCFVARLIIKLSFVWKEDKQ
KTIENMGVTLFGVMIFSKLMICQSKGIVTLLRKTLNDEEGMRNDTSQEDKNIYIDNAKYTFKVTAAITIYT
YLFLGLPSIILNYIKYLKWKRIYQNTNSTCPKPLLMVTWYPFDEDEHYFLAFGIESVSLIVAATYNNVTQTF
FITMMRFMIGKLIILQKHFRNFDTCENGGCARKSIGDEQAVLKNLKILVLEHSKIMGFMENLNESMRYLML
LEFLMNSVQIASLLMRILMNGLSLLNLLNVFALGMITNQLFLLAWNANEIKEQSTALNDALYESKWYEHS
SSVRRYIYLMMIRSQRPLTLQIGPFYPMTIETVISTLKAAYSYVTIMR
>PverOR8
MDILYLNIKALKLFFLWPNKDATIFHRILTSILFLSVVIVSYTENAAAIVYQIIVDFEDDSILSDCVIASCNFFG
YVFIYICFQMNVRKVQKITTDFKKFLKFCPVEVIDDAEKEIQMFMKMLIMYFFVGVILNDLIPVLDFRNCD
KHRLSDYYRAHDPCGLPVRTWWPFDARNTFQYYFLLVTNTYSCLNIACVVLVITLTLVGLLIHLNAQMKH
LKYKLLNIFGDNGSEKSPIGRANVEVELQSCVQYHNEIISHADEIFRSFGALLIVHVTLTPFVFGVLGYRIVS
MEKFTDKLRYVMQLGGWIAMLFLTCYYGQLVLDESLSMSEAAYQSEWYNGNPSQGKYISLLIMRSQKPL
ELRAASITVLSLNTFVSIIKTAYSCFTLLLTISE

>PverOR9
MGGKRILKWTEISMLVAGTPISPGKSYSILYYIWAYFSQASFIFFCISLSLGATSVTEDKEKLDRNEEFLLLSL
LLLWKLAISRSSRFRSILKEVGKVEAQIFLGDNEIVQIYQENTRHNYKIFFYLSCIYSSSTLGYFYMSIKQWI
LMRNWNAEEKFLLIAGWFPVDLTKHFNLAYFHQISVVFNLTLYSLALDSLIISLVNFAAVRLVILGYKFEHL
GSDVGSRTQPVYDSLRDIVIEHKKVIRYVENLNLSLKWYFFGDFVSRSYHVSLTIMNVIRHRSQAIIFYEIFT
VMFMLSQSTCFYYHANRLIIESTDLSMKIFHSCWYDQTYIVKKSLMITMARAQKPLALTIGNFRQLDNNLL
VQIVKAGYTFFLYQILDILH
>PverOR10
MYEKTKIICMPFTLDVTRFYGLFPQKGTELNPGPTFYCKFFIFALFSSLTLIGSLLHLIKTIQSGEYNYIDLDL
TYIISFATTYIFSMFFVIRIKSLVKLYSFLSDFEKFGKPRDFDNDNKILNTLSKLHFVYLECLILLSLSFSNIFKK
ETCELENEEYKLHEVCGIFTYTWMPFDIDFTPVRQLYTLWQILGGQYIYSLAGMLAWLVLETMQHLIIRIR
DVKYNFQNAFEEKNRVVRKQKIRIAMEYHVAVLRLQDDLNDCFGVFMFTHVIFTAAIVGTGIYCFLHRQS
LSSFLVCLGWFIGLLMDCFSGQRLQDESLLIAETIYNSPWYGYEKEMMRQVLFVLANSQKPMKLHAGAF
GVMDRATFLAVMKATYSYITLLGSQRED
>PverOR11
MYFHNIDHCLKMMSIVGVQPSTERGFYQYLRYILLVLSICIIWCLVLMYPYYREEKMTLLNMTPVVTSVC
LFAHSISKLSNLFFKGRQIQNLLGYIQNSFWKIEDINESEDRAYFISRTNWIKRMYHIYSFMSTNTATYFYCG
RIFLDGLPVDRNLPFESHIPFDIPFTVLFVYELIIGFRVELNQIFANGMLAYMLVIVPTLCIEMYILSTEKSLKE
GIRAFSYSATLFFEFFMCYCLPAQYLNDEAELLANSIYFSNWHLHSKHSKDLVLLLGKSHLKMEMSAGGY
TNIDLKAGFAVSTK
>PverOR12
MLITKEHFHFRSIISLEETLLHFSGLHPAKLPKLPSWRLLRPAIPSGMIVYLLLSCASAAYSYDFDLVVESIML
GLGVLRFVLKLLIFLHQRRFFFDILQAFEEEVFYSFTSADLEHVRAETSKTKLLRTMHFLSVFTYIGFLGVN
PFIFGTRVQLVPIWEPLDLGLFTFGCHLYAAVFTAYNHVSLDTIFTMATTFACCQFDVITSSLQRLDFRQEGS
GGALRGLVIRHLRVLRWLWLVLKLPPSCNWLHFSFVTTINKLVTYMALVHYVISIFVICITLFQIMKTPRSV
NNIAFLICMVVEVIRYCSFGEAIKHKNEQLSTALYLTNWEEADTKSRKMILMMRERCQKPTLLDVGGFTT
MSLSSFVNIMRSAYSSFTVLRQAYN
>PverOR13
MGNEKNIHHVHVHMKYARFLNLICGLWPIKSSGYKLKLYNLYFFITLTYYVTYDISQGIMAFISRGNFMEM
ADNLGVLIVYVAYIYKVLITKSQPVRDIVQRLGEQEKQILENADEKIKGIYMEYVKAAERVVVFYFIVGTIA
ILVYFSRPFVENYLKKPEIRFLIFPSWFPFDPNEHYWVAISIQIGAGLLGYCYIVYMGTLYCSFLIFFVGQLHI
MRHIFRHITHYIMKFSEESGIPYEEAQGYFIKICIKQHQQIIRSVRSLSESIKTFLLLDFATSSLQLSLLVYQLIK
GTEVIERFALVWFLLTLCSQLVLFYWNAHEIIVQSTGISDAVFESDWYELKLPVQKLLAVIMMRAQKPLSLS
IGLFYEAKVDAVVSLYKAIYSYVALIVK
>PverOR14
MFVNRSDFGYPENFFSVNELIQKLVGLWFPGSDYKLPFRIAYMMFVITFYGCGLAMVVCEIIYFFDSFEHIS
VFVSVIEILLSQFAVILKSSVIFFGMKKLKEMMDTLQEEIYIAAGDFNPGEFMQKQKDTNSLVTILLVTLYCS
STSGTTIVSFIMMDKWLEQDSLIGTNLTCYDFMPCPFYIPFPTDTKLWCQVTAVFMGLGYLWSSAISGSCDT
LFIGLLSCLRTQLVILCNIFRTIRERALIELDLPENYDILYDSENPILEKELYSQLRRTTKHLMALLRVKDDME
NIFTFATLGQCMASLFVIAACLFVTSSAPVGSPEFYIQILYSLSMSIQFALLCWYGNELKIASEEVASSLYESN
WYSGTTQYKSSMIITMTRMQRPAFLTIGKFAPLTLTLMISVYKASFSYYTVFKKVQ
>PverOR15
MTTIVGERIYLAFTLKILEIVNMWSEKTGSFRFVRKHYALLILIFSTTAIISDFFLQFYDEESHFTSFIESLIGGS
ALLSVIYVSVCFTAKKDEIRRLLLNLKIFENFTSAEKIAEAEKSAKFYTKSFILYGIIGNGLYSALPFMSLKDC

SDKRTRQMVRLGIPCKTIVRYVLPFKYDQSPFYEFVILEQVAVALIGTIVVMTVTMLVCGILTHIAVNLVYLR
ENIKHISKIPDDNLKDHISYCVKYHTAILEISKDMNDAFGVMMLIHITWTSFIISVLGFGIIMENNYWNMMR
FIMHLGGWLLMLFLVSFYGQILMDKSSDIAKEVYDSEWYWTSPKNRESIVLILLRSQRPIVLKAAQVKTM
SLSTFLGVLYSAYSYFTLLLKLKP
>PverOR16
MKYVEFVDFISFHIRLFKYFGYWIPDPEDSNLYKFCYNFYNKFCTVVWFFTLVSQVTSLVLNIGDMNESTD
IIYITVCCIVAFFKLLAIHDNLKSVKRLLVQLSDPLLLPKCDKHLELCKDARAFQKKLFMLFMFMGGQITFS
FAAIPLFSDGRLTVLPGWFPFDWKSSPYYEMVYVYQSLMLLWICYLSSNADTFTVGLLMQIGLQCDYLCV
VLDYLEEFHVEDGVLVSDDDEDEEVRRRKRDPEEFSRMMTENLVLCIKQHKFIERLAADVENIHRVSIFILS
LGSTLVLCTALFQMTQITLSEDPAKFMMLVSILICLLIQQLMHCWFCNEIILKSGRIFAVAYNTPWVDCDLRF
KKLLLNFLTQTIKPIEIKVGGLFSLSNNVFISIVRSSYSYFTLLRTMQENSSTDN
>PverOR17
MSLGEGDSRISVKILRPCGFLKIQTRLDKLIIISSFILVVIFDVLIIIKCFYVDGTIENFVTLSEGAGSYAQMTV
KLWTLISHNEDLREMFFIIDNKFWKHDSFGPETRDKLKSHLRIVDIFIKALAYSALFTLTIMFLKPILLHKLIY
ECWLPTGDIWFWVGVSLQIETLIYCLFVVIGFDALFVTLVIETTMQFKLLNVAFSRMRNGDDVRMCVQHQ
CFLFQFVEKLKKLFSIFFLVEYFDTIIVVCMQFLIVMDSKQNIPIKLKGFFYITCLLLQQAFSCFPISLLRDESE
ESTNVIYNCQWLKENDSFRRKLVLMIMRAQRKISVKAGGLFEMDRTAYINVCRSIGSIYTLLSTIEQRSIV
>PverOR18
MYRGIFSSTKWVPEDFFSWMDSFYELKGLHPPKNKIWRIIYLSLSVPNILTCTVLLISLEWFSLFWTEQDFK
SKMKNISSATLNTIVVFRIAMWFLTRRRMEELKVLIRRKSFDFECFRISGIHRESSYETESMSYQELSQFWR
NVDVVYQEGSNDLQSFANVVMFYTRFGCCIIYISIAVICFMTYAMAFAYEFNTENGVYDALNPYLNKTSRY
RTYQYPIYFPFDVSFDGYYWLGFAYNCYANIGSILLFLPIETLITCSIIHLISQVIVIQEAFTYVEGNILEGDGL
LEALIIKEFRIVMCINELQEIYRAIQILEDVLSKQLLVQYSFATFILCSVCYIIPLVDTWIELLVYMTFMIASLG
EVFIFSYCCQTLAVKLQEVAISVYGLDWPNYSLKMKTSLCFLLKRLQKPANLTAGKIINIDLLFFIKVVQKS
YSFYTLISNTESKK
>PverOR19
MTKQFDVISCVIHQIRLFKFLGYWKPDGKVPYKRIYKFHTFFSSSILVTFIVSQMLYLHFNRTNVEEFTSTLY
VTVTFLVVLMKIGSIYKNTGRIEAYLKTLDNSPLTRVKCKRDFNLAKNTKKLSLTIFFLALYFGMQTVLFW
SIIPIWQGNGYSTIAKGWFPYDWQSSRNFVFTYIFQFIATLWDTLACLNVDSFFSSLLQIGLQCDLLCATLN
NLDQFYSEDGVLYERNDDQVLTIHHHRFSEELTNNLVVCIEHHVMIVRFVKEVDDIYVLSVLSSFFGAAAI
LCTCLYRLSMTQIGSMEFGLLFFYLGSILLEQVEYCWFGNEVKCKSANIMDAAFNTPWVDCDSKFRKILL
QFMTLTTKPLVLRAGGLIDISVTTLISIIRTSYSYFSVLKQMAD
>PverOR20
MTVFKVDNRNIVVYNITVLTFYGIIPPNFENTQFLVKILHFLRIFLMGFICIGLLICQTIHLFLAFGGQIQELVT
AAYVTLTNMTTGIKLYRVYQTRRRIMHLVDTMNIRVFQPRSQHQVGLLKSYARLSNGVNLVFVVIGSMVL
LFMSIYPYTMTGEPTLPLPAYIPYSTNSSPFFEMTYAFEVSGIVLGAIAGINADGLLIGFLIFICAQLAVLNDSI
VNIRELSLIGAEREGDISRGVLGGKMNAMLVECVDQHRRISAFKSEVSSIFGYLTLTQFVISVLTLCTNLFEL
TMVTELNAQLFFLVFYLIFLLIQIFFVCYFGNEIISKNFQLTNSTYHSDWLNGSVNFRRNLIIFMTRSHIDMK
YVAGNLFTLSLETFVKIVKSSFSYFAVLQHLGDSEE
>PverOR21
MSSEFPIGSLGKKEYHFRNTIRTEENLLLNLSGLHPQNLPSLLSLGVLKPFTSLALVTYMVVSICMPVFFYDF
DLVVESLMLGCGMVRFLFKISVFLHHRSTFAAMMEDFDDEVFYSFTVEDLGHIKAEVKRTRILGLMHFSSI
LVYTIFLAISPFIFDKEFQLVLVWKPLEMGVVTYTCHLYALCFAAVNHTCMDNVFMMMSTFVGLQFDVIV
RSLERIDFRKHQEAEVVLKRHIIRHLRILGFVNKMNCLVSYMALIQYLISILAICVTLFQIMKNPQDGSNITF

LLCMIVEIILYCWFGEAIKDKNTDLSTTLYLTNWEEANVKIRKLILIVGERCKKPIKLDVGGFTSMTLNSFL
NIMKSAYSAFTVLRQVYN
>PverOR22
MQSKLNYIDFFYFHISTFKLLGYWKPDERMKYKKLYNFYTLVCTTVWVTFLVSQYIYIIGSFDNIDELTDVS
YVAITCSMDLVKMLAIYRNMDLIKSLLGKLNQPLFQPRCHDHLLMAMSVKKFQQTLFFLGCYFGVQTYLI
FSAIPFFRKDLVPLTQGWFPFDWSKSYAVVYVFQNVVILWNTFIFLNLDTFTSGLLMQVGLQCDYLCLTLS
GLGRFRSFGGVLCEVTEDENEMLGTEVYSGVMSQNLNACVEHYQEIKRLCKDIETVHHTSLFILLMGGS
MVICFDLFQLNMVKFGGIQFYLFLSFLLCMLMEQFIYCWFGNEIIHKSGNILTSSYETPWLKCDRKFRKNL
LIFMTQTRNPIGFKAAGLLLMSITVFVSVLKSSYSYFTLLKTMQENQMIE
>PverOR23
MINLHKYIAQEVKLLKISGFYPEKIPTVMHKVRAFLVILITNFCTILFYLDFNNADQNTFEFTSCTLYAFGTS
FVSWKFMAFIKTQRNILKLEEILTNPILCEFSETNEQIFENIVNSRFFIICKGIGVALSTIVNCMVLERGFKREL
YLPMKLYGSLENDTNCLYHVILQIIFMSIVTCNYVLIDVLCCKMISIATATLIILENNLKHVPYGNQDFQSLK
TILKKNIILHREIIRYISHMEYTFSYVILTIILSGVFVICICGIIILKIDLSLAEASTVVLFFSCLMNQLAIYCWFG
QQIMTKSVEISTGSYMTEWYTADIKIRKYLVIFMERVKKPIIIRAGYFFPLNVETLASILRSSYSAFTVLQQF
YKD
>PverOR24
MTDKTDSIKVMKNSLNFLRIPYIFPKAEDIDNPKKNIYIRYILLNLSTYYVPLGAAFHLATTIKQGTYINIDK
DVGAIMSYHSALYFNLRFFFNIKNLIKLYKQYSDFESYGVPTKFEKMNKLLNKFSMIYFCYHILIMAGITTS
TLFLVETCEEQNTNRNTTEVCGLISATWLPFEYDYFPLKQLVYGYQVYTSCMIFHTAGILSYTMMETIEHLI
VRFEHVGDVFVEATAQKDPQQRREKFNAAVRYHKVVIEMGERLSECFSPCMMVHISLSGPVLGIAGYRFM
TEILLDATSLFFGWMISTFVVCRGGQRLTEASTSVGNVIYSTDWYNLEKDLQRDLRIVMMRCQEPVAVKA
GPFGAMTYTTIVTILKTSYSYVTLLKQTM
>PverOR25
MILNNFGFKFLSTKDHLKVLFWMPKLLLNLIHLWPVDDNPFFSRVFIFIPTTTCTLIGIGLMIRVVIDSNDFD
KTIENMRSITTVAETILKIFSMYYYTKSIKLILDDIMEKSWPYDLMGTDDDMTKSLRELNKNVMRMMATL
LFCAFLYALLCCFIPVVADEKMLPFPAYYPFDHLVFPTYLLMYMFHVISLFYIMVPGVIGFDLLLLAICSSIV
GQYKILQKGFAIFNTEKMYQVNRNLRIVLEDGLEDKYEIKKEYLVRCIRHHQYLMKLLNKTNRVFSFLEM
VHLVISALSICIGLVLTNQADTTTSQYLTATYFTGCILFQLFLYCAIGNEVHYQASLLPQYIFQSKWHTLSDT
GLKKDVMFILMRAQEDQELTSYQMIKMDLLLVVRVVRLSFSGYTFLSTMKNELKKMEGN
>PverOR26
MYEKEFRNVFRLLNVVGMNPTKSYNKPLIIFNATLTLYVTVLIFMNLILKRELVTVESIGVFSQVWLKFVILI
TKKQKIKEVIEHTQQFWNEDPQGSNNASMLKSLDKLERTFLIYISCSTCMFLFKPLLVRGSTIYYYYQIPQIP
FLLSYGVEFYVTLVTMSMVIAVNIFISISIVIGAGQFSNLNARIRQMDVGKAAEDGGMENVMGVLKRNVA
YHNYLIEYVDRLDDIFSLLFMVLVEIITALLCMNMYVLSLPNTTVVDYIRCGTMVCAFTTEFLFLYGVPAQ
KLMDEAEQVANSAFYHCDWYLPNILPIRKSLTFMIHRSQKTVGLSAMGFIDINRQTIVAMIKTAYSFFTFLQ
TVESSDTPK
>PverOR27
MTENYPIYDSFKYNVLIMKLCAIYPPDSWSNSLYKIYAYSSYITITVLFPILAAIYLFSAEIVDVHQTCWNIIL
TIETGALIVKVFVFVIKPQQVKKSREDLGKDIFILYAPSQEWIIKDTIQEYYTVFIAITLFSVFSFLAHIFLTAVS
LERKFPVNIWLPFDGLTDTKIYVMVFIYITFGKLIVSI
>PverOR28
MQPQIFKRERDDDELDLRDIVKINIDVLHFFGNMYPEFDSFSVEFVYILRVSIFFGFFFMGIVLAELANWYL
SIGNLQAMVSASFLTLSNIVSIIKFYAIFRHQDKIFILLESTNRKEFRPKNREQTSILREHINVTRTISMLMYGG

CLFTCAFWAICPFTDEDGPYLPIAAWVPHEPSKGTICFVLVFAYEILATIVGGITDMSADCLIAGFLIVVCAQ
LKILNNSLVNIRQFALQELHNNGSHYDDDEHDETWTMLQAVMDDKLLECVAHHRCILEFAEDFTSLFSYSI
LGQFVVSVIIICTTLFEITMVSFRSVKFFSLILYQYCMLMEIFILCYFGNEVILESNKLTNSAYHCNWRPCSLQ
FKGNLLFFMTRSQRVLKLYAGGFFTLSLDTFVRILKSSWSYFAVLIQVTKGNSDI

>PverOR29

MSDNYADDFFVVNRWILRWASVWKPENGNKNIQTLYMIYAVVVFVLVDIYFIGTEFLSLISKFGNEYEFMK
ICSFLLTHLMGASKVLFFHFRGVDLRKIMSTLESAQFRYGSCPEKGFFPGETSKHFKRVGIMCSMLFLLMA
HVTLMSSYVPSLVAALQHVEGNPEKPLPDRMPYYSWMPFRFDTTLLYVIALVYQATLLFFQAHCIVGMDT
LFMNIMNCIGMNLEIIQGAFFTVRERASEKVNGPLLTPDGIYNSEELKTALNDEMRKIIKHLQTIYKMCDDL
EDLYKFLTLAQTIATLFILCSCLYLVSTVPATSEQFLMSEVVYMIAMGFQLILYCWFGNEVTLKADELPFHI
WQCDWISADTKFKKSIILTMARAKRPLYLTAGKFAPLTLATFVSIVKASYSFCTVIKKSSH

>PverOR30

MEILYLNIKALKLFFLWPTKGSRLFQRIIPSILFISLVTSCYIDIAAASIHQIMVNFEDDGVIFDGIISTANYMG
YILIYISSQKNARNVQRLTGDIEKFLKYCSVDVIDDAEKEIQMFTKGLISYFFGGLILNNLIPLLDSKNCEKQ
RSSDLYRAHDPCGLAVRIWWPFDGRKAAQYYSLLFRHAFVSISVAFLIMFITLTLVGLLIHLIAQMKHLKQK
LLNIFSDDIDESIETDINDVEAELQLCVQYHNDIIRQAYEIFRCFGVLLIVYVTFTPFVFGVLGYHIVSLDRLT
DKVRFTLHLGGWIAILFLTCHYGQLILDESLSVSEAAYQSKWYNGHPRLRKYISLIIMRSQRPLEMKAASIT
GLTLDTFVSIMKTAYSCFTLLLTVAE

>PverOR31

MESKIPEDPLDFIYLPKNIPVVLRISMILSGLVAQVFVLVAAYDILIIGNLNTFIQYLTSLITSQVASSVSMLYG
LDIQNFVDCPKPLKESIRCFRTMSMKTRKTIEKEIVWIKRLYKASGPIFPFTVFVFVIFKVGDEDDWQYYFY
KLRSSPNALKYSLCLPPCISLCCCTWWTFNLLLYNLTHLRYRCMILDEMIGSLNTKHDYHVNDIMYQRRV
SKRLKQCIRFHIKLLWLKNTVMHQTLKYAVIANMWLVGISFSAVYFIYAEISPPSNFRMVLIIICYIVTIVDVG
FYHQRIINKAEEMYYTTCGCPWYQWNISNRRMYLIFLTNISMPFRFPTMGTFEVNNEMLLKNARMTLALI
TFYTNVKDVFN

>PverOR32

MFDQTDIKCMPFTLRISRLYGIIPKEGSETNPGIIFYCKFFIMAILSSLTLIGSLLHLIQTIQNKEYNYVDLDLT
YVISFSTAYALSISFVIKVKSLAKLYRFFSDFTKFGKPKDFDTDNKYFNTLSKFHFVYLESLICILLSFSNITKS
QICELENEEYGLQEVCGLFTYTWMPFDINFTPARQLYTLGQVLGGHYIYMLAGILAWLVFETMQHLIIRIR
DVKHSFEKALEEKDEKVRKIKIEFAVNYHKAVLKLQDDLNDCFGVFMFTHVIFTAAIVGTGIYCFLHRQSL
SSFLVCLGWFIGLLMDCFSGQRLQDESLLIAETIYNSPWYGYEKEMMR

>PverOR33

MANMYLVKKDTAFYSSLRTLRFFFVYPDKENSKFIMASYISFILSTFVFIIFILGSSIHMVMALKANAGGDIS
EDLSVILGGLGMLTNVGMFQHHQSKWSEFFSDVTDFEKFGKPTGFDKSRDRGNLLSAGYIIYCTIGSVVYS
FVAILESSCKDQEEGDHKEICGTLTPVWLPVQEISLMTKNAIFLVQYCLANYIITSSAVCCFLPFETTELLISHI
SFLKENFLRVFETDQEEIRVERLRFCVNYHTFILRMTDQLNFLVKFTLGHMSLICALVFGCIGNQIFRAKPL
GAAIFLLGYVVSLFLLCYAGQRIINESLSVVDVIYESAWYEGTIEMKKSLKFVMARCQIPSRLSAWPFGFFS
FPLFLMVSTFMKLDEYQIYRFFF

>PverOR34

MKTTNKYLKHYANGIMLILCISGSCSLIGFAYLFLAEISPTSNVRFCFCLASELYLCVVCCSLGQMLVDVSD
DYYKLLCSCPWYNWNLKNRRVLLIMLPDAYHDPIQINCLGLKTLNFPFLLQVRTGKF

>PverOR35

MSINIEVRKKQLQSLDQYISSSKEKVESILGYLGWTSNKVREKKDEISCPLNIRHKIHFEKIGKHLETCSIRT
AGYDPEEQFLSEPLHSVNSVTLDNNQKIEILNEARCSNVKFRQAWNGVDPDPMTSDRLVSTFSPDERLALY

EYCISNTEPPPTPSEFTLKLDDSEEKKVDKQLTEEELREQIRDAKRRRTKYKSVHTGRNKCYTEVMRQVIE
NQMDLYREYLVDKLRLEKEVEQKRLEDAYEQFDQPDASNIIYDFDADPNSVGSNVENQYTNFDLVYSEFT
EEPLPESSEHRSNHSSESANQVQGEQDQERRRTDRYDEHKRSSRQSTREKDGRRWSRSHSRLRKGNRTSY
EERRSRERRNGSRLSRERREPRYHSDRRH
>PverOR36
MERKHFEYLKFLMILAGIWRLELVSKSKTLKYVYGLYTVFIQMTFACFVIRLFVRLATVWKEDINVTMEN
LETSIFCITLTLKIIICQSKGIIDLIRKTLADDEKLRNSENQEDKMVYYDYANHTFKVSLAITFYTYVILGIPM
VIFAYINYMRWEKKYGGTNTTVPKPLPVVTWYPFDENKYYISAFCLEVWCMIAATTCNNVTQTFFVTMM
NFMIGRLIVLQQHFRNFDSYRFDGATITDEKVLLGALKELIKDHLGIIGLVENFDNSMRLLMLLEFLTNSLQ
IASLLVQFLMNDDTLVILHVITYIGIVTSQLFLLAWNANEIKEQSSALSVALYESRWYEHGISVRKCILLMM
MRSQKPLTMQIGPFYPMTADTAISVRQ
>PverOR37
MQILSLSVLLMQIGLQCDYLCVVLDYLEEFHVEDGVLVSDDDEDEEVRRRKRDPEEFSRMMTENLVLCIK
QHKFILKSYFRLAADVENIHRVSIFILSLGSTLVLCTALFQMTQITLSEDPAKFMMLVSILICLLIQQLMHCW
FCNEIILKSGRIFAVAYNTPWVDCDLRFKKLLLNFLTQTIKPIEIKVGGLFSLSKQCFLYRLSVHHIHIFTLLRT
MQENSSTDN
>PverOR38
MDKLFKKYVPEDCFAWMGIFLKLHCLSPPETKILRKLYTVLSPPFFLIIFGVPLICEPIQIIFSDDSVERKLLTV
GIMNLHIASVIGIVNWLITRGDMEKIINVIRRPTFRFEIFDVTDLESGYRDLEGKYRSPIPAFQKEVNFRTSKL
CSCGYMTFFVYITVTISTSYTINYYQDTYEKFNPHLNKTSVYRDFVYPLWMPFDYSLSDGHYFLAFMYQP
VGYFLLVGGYVVVLWIILDVLIHLKGQVSIIAFALTQVDCNIPKEKFENREIIQEMRIVKCIEELQIIMRLGAK
LDCLINIMLLLSFLCSLFILCCLSYLASVLSTPFQISLFASIFGATLTKVFFICYFCQEFTSEFSVVSRNIYELDW
THYSPRLRRMMVFFLARIQNPPTITLYKWVNVDMVFYLNVVRMASSFYMLISNVNTK
>PverOR39
MFFYSIRICLKLLHISGLHPLKKRTQNDKLLFVILFLALCSIILLDILKMFYKEQLEFTDYINAFSEMSMIVHA
IIRFLSLFSNRQGLNLLLTQMEERFWDYQSPEVSPEEQIYQKAFQTKVKYTFFGFLLCCAINTSFMSVLTLF
VDEDVLVYECYRPTWLPFYVLWLYQISAVNMAMLIPIICFDTLLMNFINLTYLQFRLLNRKIEAIMESGDVL
EIRKKIRLVVEHHHFLMKYTQLINISFSSCLLAYMIMIVTSMCVEMYNISSNTDIGAIIKSIVYINVAITGFVIL
FCIPAQHLVDEAEKVATSAFSCGWYLCPDFGMAVLTIMANQKPAKISAGSFVDINLETSLATIKTMVSYCMF
LRTMSIDE
>PverOrco
MMKFKVSGLVADLMPNIRLIQASGHFMFNYYADNSGAVHTLRLGYSCMHLIFCLLQYGATFGNLVLERD
DVNYLAANTITVLFFTHCITKFVYFALRSKLFYRTLGIWNQSNSHPLFVESNNRYHALSLKKMRTLLICVM
TTTVLSAVAWTSITFMGDSVHRVKDPDNKNETIIEEIPRLLIKSWYPWNAMSGMTYYISLIFQIYYVLFSLTH
ANLLDSLFCSWLIFACEQLQHLKEIMKPLMELSATLDTYVPKSADLFRAPSANFQDNLIENDYNTKNEELN
LKGVYSTRQELGANFRSGALQTFGQGGGGVGPNGLSKKQELMVRSAIKYWVERHKHVVRLVTAIGDAY
GVALLLHMLTSTVLLTLLAYQATQINGVNTYAASVIGYLVYSLAQVFHFCIFGNRLIEESSSVMEAAYSCH
WYDGSEEAKTFVQIVCQQCQKAMSISGAKFFTISLDLFASVLGAVVTYFMVLVQLK

GR

>PverGR1
MHGFDPHRLHKNPHQQRNEPFKKPNETIQVRGTPASKDTNQPDPEVLHLHDNFYHTTKSLLVLFQIMGV
MPIEREVGKTTYRWFSKANIWAYFIFSIETVFVSIVFKERLLLVLQPGKRFDEYIYAIIFLSILIPHFLLPIGAWT
NGSEVAKFKNMWTKFQYKYFIVTGTPIIFRNLSVITYTLCTVSWLAGIVLMLAQYYLQSDMLLWHTFAYY

HILAMLNCLCTLWFINCTAKGRAAGWLAENLHNALQSSDPAVRLAEYRDLWVDLSHMMQQLGKAYSGM
YGMYCILILLTTIVASYGCLTEILDHGLSFKEAGLFLISFYCMTLLYIICNEAHYASHKMGPEFRERLLNVNIG
VVDIRTRQEVNMFLTAIDKNPPIMNLNGYANINRKLITSTLTSISTYLVMLMQFRLSLMRNASVASRKATIG
GNTTSIT
>PverGR2
MIVALTIMIMSCDSTEKSGLQVIKTCYHLHDVVEHPVVKEQLLLLAEYARQWRPVFSAAGFYDIHQGTLSS
IFSAFITYSVIIIQFNMVLVDEKANATTTTGS
>PverGR3
MGDFLIIALSLIVSSRLKQVSMGLESSLRNKRFTSSISTNQETRGRFYTRIREDYQRIAAICERVNELLSMIIN
SYVANIFFLLVQLYNSLKPMGSVIERFSYFFSFAVMIFRLVLISIYAASVNEESKRFLLVLNNVPSELWHHEVE
RMIGQIDFGGAQLSGYGLFTIDRG
>PverGR4
MRLTYSRRCILFIEPMAKLATLFCILPSYSFDKQKLVHEKLFIVYAAIVFATMSSAFGYVFYLRLESVYPYIH
PVLIYMDILRDGVGVLLAFLTAFKSVWKRKAWQRLFLNFDRLEAPENLNTKYQREKHFFTNANLLFFLNI
ALYSVVIGSTMVIEGELFLVDIKYNWNFIIQSYTELLHVYLMYNLVLAMMNKYEGINDLFEARCSLEDLIP
KIDRVENLYYIMDDVVELFNVLCGWGILLVILHRVIIVLYALSAPLAPSYIIDGKTLKRSPDLLLINICFSLISL
FQVMVMICCIDMTKKEPLKLVKTSIMIKKSLPVDSKEYARLELFKSRVSEILPSFTAAGFFKIDRSTVLSVIS
VITTYFIVVVQFYLSSGGS
>PverGR5
MVAEDTSNEKPKSNALIRTITGLSLLAYFAFVIYVGVPLILLNALIIQIKCFDEVITIAYNMKKMPDLPHFRTL
NWYFVFVANYFFFGETFAKHFEPYVRKYYSLQVLAAYHRFFSFCWYLAGVIWFLSLLRKELIKKQFSLFF
WTHFLIIIISLQSYTLVQNMFEGLIWLVMSLWLIILNDVFAYVFGKFFGKTPLIVLSPKKTLEGFVLGGASTF
VLGAILAYLFCQIQYLVCPQRYVDAGDTILLDTNCTRSYLFEVRNYAIGETGFSVRMYPFIFHSLIMSVFAS
MIAPFGGFCASGFKRAVNVKDFGNLLPGHGGFMDRFDCLFLMATFVNVYIKAFIRNSDVDKIFQRILSLGK
EGQMEFYFSLKDSLVDRGLLNIYE
>PverGR6
MSCDSVEESGKKVISNAYILHETVEDPRKKDDLLLLAKCEEYWRPIFTAAGFFDVNQSCISSIFSALITYIVII
IQFNMVLQDEETTMDNNVTVATTGT
>PverGR7
MTKTKDNFTAGNGFKQDYKFLVLCLDLGGYIGLTPSPVNFTGLNKTKLKKICQIILHLLLAVYACVSWKLQ
WEIITTSFNSMFRTLEISTSAIGVSYVFWSSAYLSLICRNDWTRLLSKMQQLEHEMAASKAGGSHHALRAR
VVFVYSFLFSFHTTRECVWYSNQAYNSIGAFSFTFITHFYLTSLLVQHVVFSDWLANRYDTLNEELARWGR
TPHRLGAELEEFTKLHKLAGALVVAHNRIFDSLNFVQVSVYVVFILNTLAYSVDGHQSTMGRKILDLVQPL
FHSVSLLLFTEACDAVEKSGRRIVRTCYSLHKNIQEDALLKEKVKVLARYAEERRPVFCFWNFFHLNRSSL
LTFFSAIITYTVIVIQFNLSVKKKIVSNSRNATLI
>PverGR8
MDVNRDVRIVKTFLKCGTFLGVSPLINQKEKSTDKSHVIYKLYVIALFNAVIFGSIYSILNRYNGFWKENTI
SQMILEAVEVLSEMMFMVCNFLGALKYGPNWKSVLASLKQVDRALGLKKSAEKREYGYMCKIFIFHLAY
ISLHTYELCMWTAQSPTTIVNEYGYILYRIIMYYQFYMTLFAVEIISVLHSRFLQLQIILDGISSKRVIIISQNES
QYVLGKLLKLRKLYGILYEMQQNINFIFRYSIFYGTLSNILVILGDLNSIVEFAHSSDETFNIGVSFINFLYAFY
YSSASVSIVMSCDKMEKVSTKMAQTCYLYKDIFEKTTLSDEFVSLAEYVKKLNPTFSAAGFFRVNQQLLSI
LVSAIITYFIIIIQFNLRF
>PverGR9
MLKTVVLSRENFQFFQPILTVSRIFGLCPVSCKYDGPRVIIGWSNLLAIYSYLLSIHFIIVSIIGLVNDIDEGDS

KSFRMKMRKGKFITCCDVIIIILIVIFGTITIPCRMRRFMDILKNFNKTDSVLGLPNLKGYRRQSIRMILAVYC
LFTSVVVFDIFYWTKSSCRHEKTGGQYILRYIGFYLLYYIMITLELYFSHLVYSIHIKLALINEHLVIGNTVNN
SERDVDVISSRIDDFHHQICRCTGPKDFETIFKIKIPRTRINNAQRIEILRNLYKQLNNGVKIINETIANGIMLI
MLSCLVHLVVTPYFLVAELIKDKSNYFFIFLQVLWIITHTCRLLIIVEPCQACSLEGQRTTMLLCDLLGLSST
DESFRKAVQSFSIYLSQNQIKFSCSGLFTIDRSVITSVAGSVTTYIVILFQFNNN

>PverGR10

MVIEKNISDSDFGEISANKQHGATLHYVYPLNRIIGKPLESQECPFTESHLKPTNRHTNSMEMITQPIPKKSR
IVLDNKLESSAMNLSIKFLLKVGQCFGFFPLDLDTSPPRFKWTHWRVAYSASTVLLFSLEVILVIHETGHHE
LKFTSIATFLFQFFALLEFILFLKLAHQWPSFVKEWSKVEFNMRKNYEIPCNLRHKMSWIITPLLAFAFVEH
FLVNAYKLKNCLDAQANPTEAFRAFFTSSYEHVFGVVGYSFWLGLLVQLLNFQRTFVWSFVDFFIMMVG
LALSFRLQQLARRVKLASRSRVRLVVSPGIPQLSLLLQVKQWRVLRLDYLALVGLCEYLNQHLSTIIIVSFIS
NSYFVLYQLFNTLRKMESTVQKAYFYISFAFLLVRVLGVCVLGGAVHEEWGNIRFHLGHVGSLAYNEEVE
RMVHHVTTWELSLSGKNFFFISRELILSMAAAIVTYTLVLIQFYSVDGNQK

>PverGR11

MFTRILAMVSVSLLLTEITEGAPAVASSRQFLSGHPGFIPVYIRAGDTPLEDINPELAEAFNSYAQRHGRLTY
RRSLEDKSGETGGDEEFPDFAEVGETSLEEDVKPEGDNNKISGSQEASGSHIQKIPRN

>PverGR12

MTTRKTKFVTCCDIGIITIIVCFGILTLPFKVRKFWKLMECLNQTQKTIPIKASRRYALSSIYFLAIVISAFMVL
FIFDIIMWYHRGNNNSTYLKEYSTFYFLYAMVVMKEVFFWHVIFLIKIKISLLNNKLLDIKNKAISTDIHFIG
LGGNLNTIDNGYKCGNSKDLGGYFLLTKVHKDSFNVYTR

>PverGR13

MVCFLVYTTVSILNSCSWCLYYVQAENFTPGLRMGRTISSLVSPLYNLIQTVFLVMACDRVETSGRRVINAC
HMMQRRIDRSGLGEELIYLGKYLKYLAPKFPAAGLFNVNRKILSSLLSCTTSYII

IR

>PverIR1

MLSKILLCVIILEVSSGETENNTEIIEILNALIVKENAPTKITAFICWPKNVQFQLLKRFSGSGFPTKLAQSGD
DIRDDSFPSEHHLIIMDLACENGKAIFQEVKQSDTAPSRWIFWGNQDESITMSLLNQFYFKIDSRVFLISEKS
ESYRIKSIYKLEENSETFMENDVANWGPRRIGFTSFNDLSGPRNRTNLMGLTVKVSYVITNNDSLNHLEDY
RDKHIDSISKLCWILIHHLMTMVNATPTAVFRTTWGYRNASTNMFSGVIGDLQSGEAELGGTASFFTVDRI
DVVDFVAPSVPTFMRFIFRAPPLSYTTNIFTLPFETWVWYCSFILIAVVLLVVHLIVMWEWKDPIFREYIEEK
HGNMTPLRPGFSNVFLMEIGAVTQQGTESEPKSIAGRIATIFTFIALMFLYTSYSANIVALLQSTTESIRTLED
LLTSRISLGVEDIVYAHYYFENAQEP

>PverIR2

MMLSHLILCLLILPVSIRSSTANKTAEFERTLSFFKDFLRKLGYSRLVAYVSWSKAECTSFSELLSSMSILHAI
IKNNVPVSQFIKRNERIVIVLEMKFKDSLMMLQDPKNYLLFREPFIWILLSDPDANSSLHNVFMPVNSIIYK
VGNTKSSRIVVEALYKFDRTSTNFLTNRIAEWSPENGFRYFRKLISTTNRTNFMGTPIRTSYVIFDNDSLNHL
SDYRFIDTDKLSKINYRLIDSLKDLLNTSRVNIWTSSWGNKNLTTGEYSDGIFKDLLSNKADITGTTAFISKD
RLDSFTYLVPTTYEEIKFVFRAPSLSYTKNVYTTSFSPMFYLSCLSILVLGGFLLYFLELVEYQCGAIPNRMT
LMDVVATQICSITQNSVIRIPELPATRIAVFFNMIFCMLVYTAFSAYIVILLQKTTDEIKDVRSLYDSKMPIGV
QNTPYNKYYFSTPNLLTNEHYRKLFYENRLGGSQSNFISPEKGMKNVQGGFYAFQVEQATAHYFIKTTFN
ENEKCSVKSIPTIFVGFQPYLVIPKNSSYYNHFQVGFRRLFETGLHQREYVRFFTKEHKCQGAQRNYDSVR
LMDCYFPFLIFTFGCTLSIFVLVTEKIIKRIGKKGLMQEVCRTKDGYRKRITQPLDFIN

>PverIR3
MPLLQFIFLCICVCLVIWFKCKENRKLKMHSPFGLLGVQILTSNSSSMWTVIDHGKVLANAQIQRMTQLA
KELKGKTLIITTLQNGDLSGYEEKNGTLVGTGLAFDILHMFQQKYGFTYKIILPEDNVFLQEPSHRGAKNL
LQDEAADIAVAFLPIVNSFRHDVTYSTYFDVAEWSVLLNRPKDSATGSGLLAPFTTEVWIVIIFSVLIVGPIM
YLIVLIRARFSETHEESRIYPLPDCMWFLYGALLKQGSVLNPVTDSSRIIFSTWWLFILIITAFYTANLTAFLTL
SKFTLPIKGPSDIGAKGYKWVSNKGNGIRDFIYSETHDNWSIRNKLVDRIGRGQLYSDRKDMDILEEYVAS
KKMMFIREQSIARHLLYDDYKAKTKRGMRENERCTFVIAKFPILEMPRAFAYRRDFKYKKLFDNSIQYLV
ESGIVPFKLRENLPDAEICPLDLGSTERKLRNSDLSLTYFIVAGGLAFAICVFLIEILWRLGSQRSEKFRSVRK
QHANVRTWMEKNENLMKRTQGSKFSIATPPPSYQTLFRPPFFFSYSDGHKKNINGRDYWVIQKNDGFREII
PLRTPSALLFQFSH
>PverIR4
MNGFRMNIIFILLLFFVGIYGQNLSRLKLVVLKEDGQDEVIQWYNDIINSMQNTEKIDSVLVDLVDDDTVN
KEKICEALSDGGTILLDLTWFGNDIGRTHAYDIGVPYIKIDVTISPLLDILDKYLDYRNSSDVVIIFENPSYID
QALYHWINTARMRMLISETLDAETAKKLKGVRPTPSNFALVANTKNMQRFFEVAIRENLVELPERWNLIFL
DFNYKSFDQSLMKNQPINYLSLDTNICCKIQSLPNSCECSDTFMMQKEFLRAALKVVSTSAQEMMRNGLL
DSSLACNNNDSSVKDDIDTQFQNILKRELSGQNVMYLEKSIMRMITSGFIEIGSDSNTNTVAKYESGVIRPE
KNATIKPIKAFYRVGITHALPWSFKTIDPDSGALVWTGYCVDFTKKMAEMMNFDFEFVEPKSGTFGEKIN
GVWNGVVGDLAYGQTDLAMTAIIMTADKEEVIDFVAPYFEQSGITIVMRKPVRKTSLFKFMTVLKLEVWL
SIVAALIVTGFMVWFLDKYSPYSSRNNKNAYPYPCREFTLKESFWFALTSFTPQGGGEAPKSLSGRTLVAAY
WLFVVLMLATFTANLAAFLTVERMQAPVQSLEQLARQSRINYTVVKESDTHQYFINMKNAEDTLYRMW
KELTLNASTDDTRYRVWDYPIREQYGHILLAINDSNPVADAEEGFRNVEDHLDADYAFIHDSSEVKYEISR
NCNLTEVGEVFAEKPYAVAVQQGSHLQDDISKTILTLQKDRFFEGLHAKYWNHSSKGNCPNIDDNEGITLE
SLGGVFIATLFGLALAMITLAGEVIYYRQKRKTIDFKKKQAKGQLPEKYFKQNRMITIGTSFQPTQFNQKA
VQDQKELKLSHISLYPRARNRITQVE
>PverIR5
MKMGLNKIWLVFLSFLLENCQGETTQNINVLFVNEENNGVAEKALDVAMTYLKKNNKLGIAVDLKKVV
GNRTDSNKFLEALCSTYNSMLSTQTFPHLVLDMTMTGLGSETVKSFTQALALPTISASFGQEGDLRQWRN
INESETDFLIQISPPADVIPEIIRTIVLNQNITNAAILYDSSFVMDHKYKALLQNVATRHIITPIKEVSQLAEQLT
QLRKLDLVNYFVLGNLKSIKNVLDAADGLNYFNRKFAWHAITQDDGDIRCTCRNATILFAKPLPNALYQD
RLGAMRRTYQLNAEPIVASAFYFDLILHSLMAVNEMISDGSWKSGGGQSSEDPTYGPFSVVSNGWSHMEF
QMQLTAIGVRDGASDKSVNIGAWWAGFDNNLTLLDAQAMGNLTADVVYRVVTVEQKPFVFRDESSRSGF
NGYCVDLIDKIADILKFDYEIVAVDNFGVMDENGKWNGVVKELVEKRADIGLGSMSVMAERENVIDFTV
PYYDLVGITILMKLPESPTSLFKFLTVLENEVWLCILAAYFFTSFLMWIFDRWSPYSYQNNREKYKDDEEK
REFNLKECLWFCMTSLTPQGGGEAPKNLSGRLVAATWWLFGFIIIASYTANLAAFLTVSRLDTPIESLDDLS
KQYKIQYAPLNSSSTQTYFERMANIESRFYEIWKDMSLNDSLSEVERAKLAVWDYPVSDKYTKMWQAM
KEAGLPANMAEAVERVRASKSSSEGFAFLGDATDIRYLELTNCDLTRVGEEFSRKPYAIAVQQGSPLKDQFN
TAILQLLNRRELERLKEKWWNKNPEKQNCDKIEDQSDGISIQNIGGVFIVIFVGIGLACVTLAFEYWWYKY
R
>PverIR6
MEIIRTVFLLTSLSVFLSTGQYVEVIGDLIETKDKVMITEYSCNTKESIKLLKLLVKHRHSTRVLPVNYSNLQ
YPLPLNRQLSVVNLDCVASSVTLQNANKLHLFASPLQWIFFSETLDTTEIMKKYFSQIDILIDSDVTLLHPN
KNGSISVKKIYKRHRRSSIVVESIVEWDEMDGFGEYRNETPVWRVRNDMKMTQLNACIVITNNNSLNHLT
DKRDKHMDSIAKVNYVLVEHLADIVNISLRYSVQSTWGYKNNKSEWSGMIGELTRNEADIGGTALFFTSD
RIDIIDYIAMTTPTRSKFVFREPKLSYVTNVYTLPFDDYVWASVISLVILIGLVLFAISKWERTKRNNPMNLK

PNASDLHDSVSDVALYSFGALCQQGAPSIPSSISGRITTILLFISCMFLYTSYSANIVALLQSSSTSIQTLEDLL
KSRLQVGVDDTVFNRFYFPNSTEPIRRAIYLKKVKSGKKDKFMTMDEGVRKMREGLFAFHMETGPGYKL
VGEIFEETEKCGLKEIQYLQVSDPWLAIQKNSSYKKFLKIGLRKIQESGLQHREVNLIYTKKPICTSRGFSFI
SVGLVDCYPAVVVLAVGLILASLVWILEIFTYYRREISFQIKTMIPSLIERNRNITHNELQYLD

>PverIR7

MKHLVFGIEIRKTTVGWGQWKRSNLGKEQCISLSIMWIYNTLAFLLYTQSIFSVSVYCVTLYNYFSQYREF
TLKESFWFALTSFTPQVGGEAPKSLSG

SNMP

>PverSNMP1a

MQLPLKIAIGSLSALLLTIIVGFIAFPKLITSKVKGMINLGPGTDIRDMFLKVPFPLTYRVYIFNVTNPDRIQK
GDMPVVNEVGPFCYEEWKEKMNVEDMEADDTIAYDPKDTFLKKRWPGCKTGKEIITVPHPMILGLVNTV
ARQKPGALSLANKAIKSIYANPSSIFITTEADNILFDGVIINCGVTDFAGKAICSQLKSSGNLKLINGDQLLFS
LLGPKNATLNTRMKAYRGKKHFQDVGRIVEFGGAKNLDVWPTDECNEIKGTDGTIFPPFLKKEQGLVSYS
PDLCRSLRATFVKDTVYDGIPCAEFTATLGDMSKNEDEKCYCLTPDTCMKKGIMDLYKCAGVPVYASLPH
FYGTDKSYLDGVNGLTPNKSKHEIKILFESTTGSPLYARKRIQLSMPLEPIQKVELFMNFTPTVIPVLWIEEG
VELNRTYTGQLKSLFTMKKIVGAFKWVVLLSSLGGLAAAGYMFYKNNGKIEITPIHESKRDGISTIHSLEG
QVNHGMSENSIDKF

>PverSNMP2a

MLACSRFFSNKVLAVLTTILTLIFVGVLVLAFYGIPVIINKSIHNSVHLEKGTIQWDRFVDLPVDILMKVFLY
HVTNPDDVLNGAKPIVEERGPYCYKQNIHKNILSTSSSQDTVTYEQNFKIEFDQEASGNLKESDKVVIVNP
VMLTLYKLTSRLERLVVFGCLDKIFPKEYIGVFIEVDVKTVMFDGFAFAQRSEDLGPACNIVRNQILDKTLP
MKNVERITDDDGILELRFALLQYKIRGPDGNYTINRGIDDITKLGHIIKWNDETELPFWGRMQSINNDTCK
KVRGSDSTIYPPQVDKTRSFDIFSTDICRVVEISFQRTDTYNGIDAYRFGITKNTFRSATTNPENDCYCIKQS
AGIDGEPSCYLDGVLDVYPCFGAPILLSFPHFLYADESYVDAIEGIGPSDPDIHELFLLIEPNTGTPLQGMKR
VQLNTVLMPMQNIPGTSKISPLVMPILWLEEGVSLPQNLIDELNSHYFQTVKLVEGIIYGLIAVVAASVLISS
GFLI

>PverSNMP2b

MVFNLKFNLSKKTLKILGCFGLFLACAGVYFGYKALPDIVTDKIWEMKVLKENTQQWDMFKKMPFPFTF
KVFIFDIKNPDEIMQGAKPTIKEIGPFVYKVYKWNSDIKWESPDDISYFAYTRFEFDEEASGRFTEDYIVTIL
NTPYLGMLLKVADIQAAALPMVEGVLGDIFKENDGLFIKVKVKDYLFQGLKMCENEGKDGDFAAGLVC
KQVIAEAATSNNLRVENNTILFANLHYKNNTHLGRFTIKAGIQNHNEIAHLALYNNQSYISIWGEEKSICNK
IEGLSTTVFPVNINKDMIFESFAEDICRRMKLTYKMDETVKGLKGYKFTAANDSFSMKNENNTCYCNKKT
TLMDGKLGCVKDGITDLSTCTGGPVMVSFPHLLYADKEYLNSVEGLDPDSMKHESFVVLEPMSGFPLSLA
QRVQFNIFLRPIDESTILANVSRALFPLIWVEESLQLDDKFTDMLKNNLFKTLDMINILKWVVIASGSACFL
FAVSMAIYNDAS

>PverSNMP1b

MQLAIKVLVSGVGITISSVIFALVIYDPLIKYVIRDQTSLKKNNEIRDIYLKIPFPLDFRIYLFNVSNPMEVQD
GAKPVLKEVGPYCYNEYEEKVDVIDNEMEDSLTYNSYDIFRFNANKSIGLSENDYVTIIHPLIVAMAYQVN
RDTPALLSFLNQAIVTIFKNPKSIYLTDTVKNILFDGFEINCNVTEFAAKAVCTQIMNSNIPGLKTDPSRNNT
LVFSLFGARNATLGHTMKVLRGIKRSEFVGKVLEVDGKKEMNLWTSKACNRYRGTDGWIIPPLLEPGVG
VWTHSVDMCRNVEAKYIKETVLNGVNARLYEADLGDMQKNEDEKCYCLTPSTCSRKGTFDLTKCMGAP
IIASLPHFLRADEIYRQQVDGMQPVHEKHIISIYLEGVTSAPLRATKRMQLNFPITTIPKLTLMTKLPEALHP
LLWLEEGVEVEGEFLKLITDKLMLLNVANYGRWLAVFGGLITTGVGVYLHNKNKNSVAISTIHSGDIDREI
TRSTNELMDQMNRIQGNEKGHVNHVLSGHEFDRYM

Files S2. The busco analysis of *P. versicolora* transcriptome.

C:95.6% [S:93.6%, D:2.0%], F:1.4%, M:3.0%, n:1367

C: complete; S: single-copy; D: duplicated; F: fragment; M: miss; C=S+D; Total=C+F+M
